# New simple synthesis of ring-fused 4-alkyl-4*H*-3,1-benzothiazine-2-thiones: Direct formation from carbon disulfide and (*E*)-3-(2-aminoaryl)acrylates or (*E*)-3-(2-aminoaryl)acrylonitriles

**DOI:** 10.3762/bjoc.9.49

**Published:** 2013-03-01

**Authors:** Qiuping Ding, Yuqing Lin, Guangni Ding, Fumin Liao, Xiaoyan Sang, Yi-Yuan Peng

**Affiliations:** 1 Key Laboratory of Functional Small Organic Molecules, Ministry of Education and College of Chemistry & Chemical Engineering, Jiangxi Normal University, Nanchang, Jiangxi 330022, China

**Keywords:** 4*H*-3,1-benzothiazine-2-thione, carbon disulfide, (*E*)-3-(2-aminoaryl)acrylate, (*E*)-3-(2-aminoaryl)acrylonitrile, Michael addition

## Abstract

A new simple and efficient method to construct ring-fused 4-alkyl-4*H*-3,1-benzothiazine-2-thione derivatives has been developed from carbon disulfide and (*E*)-3-(2-aminoaryl)acrylates or (*E*)-3-(2-aminoaryl)acrylonitriles under mild conditions, without the need for a metal catalyst. The newly developed method tolerates a wide range of substrates in moderate to excellent yields. Moreover, this method is advantageous over previous ones for the easy synthesis of reactants.

## Introduction

**Scheme 1:**
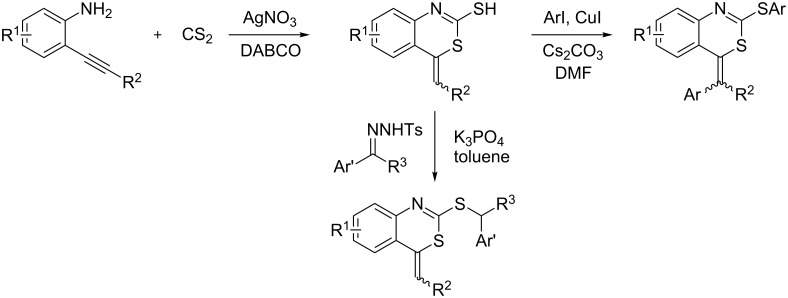
AgNO_3_-catalyzed tandem reaction of 2-alkynylbenzenamines with CS_2_ and their further transformation.

Molecules containing the 4*H*-3,1-benzothiazine moiety have received considerable interest from the chemical and medicinal community due to their promising biological activity [1–4] and the applications in recording and photographic materials [5–8]. A number of efficient approaches for their preparation have been reported in the literature [9–15]. 4-Alkyl-4*H*-3,1-benzothiazine-2-thiones are an important class of 4*H*-3,1-benzothiazine derivatives. Therefore, 4-alkyl-4*H*-3,1-benzothiazine-2-thione derivatives are also of potential biological importance. However, only a few practical routes for the synthesis of this class of 4-alkyl-4*H*-3,1-benzothiazine-2-thione derivatives have been reported [16–17]. Although Kobayashi and co-workers have reported the synthesis of 2-(2-thioxo-4*H*-3,1-benzothiazin-4-yl)acetic acid derivatives by the reaction of 3-(2-isothiocyanatophenyl)prop-2-enoates with sodium sulfide, this method suffers from the tedious synthesis of the substrates prepared in four steps from 2-iodoaniline [16]. Molina et al. also described the preparation of 4*H*-3,1-benzothiazine-2-thione derivatives by intramolecular heteroconjugate addition of carbodiimides or isothiocyanates bearing one *o*-substituted α,β-unsaturated carbonyl fragment promoted by the CS_2_/TBAF system [17]. However, both the low yields (30–60%) of the products and the substrate limitations outweigh their advantages. As part of a continuing effort in our laboratory toward the development of novel natural-product-like compounds [18–22], we recently reported the practical synthesis of 2-mercapto-4-benzylidene-4*H*-benzo[*d*][1,3]thiazines starting from 2-alkynylbenzenamines with CS_2_, and further transformations to highly functionalized 4-benzylidene-4*H*-benzo[*d*][1,3]thiazines (Scheme 1) [9].

**Scheme 2:**
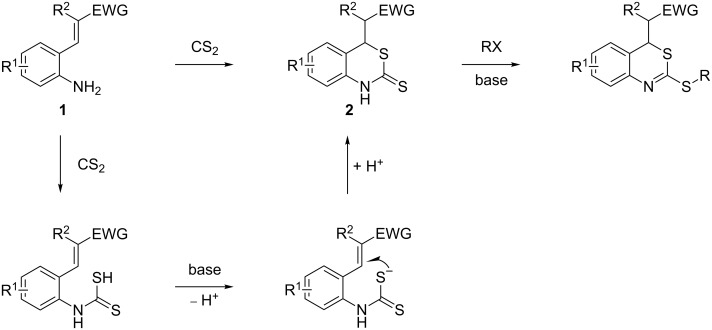
Concept for the construction of 4-alkyl-4*H*-3,1-benzothiazine-2-thione derivatives.

Promoted by these results, we envisioned that (*E*)-3-(2-aminoaryl)acrylates or (*E*)-3-(2-aminoaryl)acrylonitriles could also be utilized as starting substrates for the synthesis of N-heterocycles. Therefore, we focused on the *o*-amino-α,β-unsaturated compound **1** (Scheme 2), which would be expected to construct 4-alkyl-4*H*-3,1-benzothiazine-2-thione derivatives through a one-pot base-promoted intermolecular addition/intramolecular Michael addition reaction.

## Results and Discussion

In our initial study, we examined the tandem reaction with various bases and solvents to optimize the reaction conditions. (*E*)-Butyl 3-(2-aminophenyl)acrylate (**1a**) was chosen as a model substrate, and the results are summarized in Table 1. Among the bases screened, DABCO was found to be superior to the other organic or inorganic bases, although DBU, Et_3_N, and KOH also provided good results (Table 1, entries 1–6). However, no product could be detected in the absence of base (Table 1, entry 7). When a catalytic amount of DABCO (20 mol %) was used, only a 69% yield of product **2a** was obtained. Subsequently, the study results showed that the amount of CS_2_ had a great effect on the reaction (Table 1, entry 1 versus entries 8–10). To reduce the amount of CS_2_, we finally chose 4.0 equiv of CS_2_. The results also suggested that the solvent was crucial for this transformation. Low-polar solvents such as toluene and CH_2_Cl_2_ inhibited the reaction (Table 1, entry 11 and entry 12). Among the polar solvents screened (Table 1, entries 13–16), DMSO was the best, affording the desired product in 88% yield (Table 1, entry 13). When the reaction was performed at 60 °C in a sealed tube, the yield of product **2a** decreased to 65% after a similar reaction time (Table 1, entry 17).

**Table 1:** Exploring variation of the base and other conditions for the construction of 4-alkyl-4*H*-3,1-benzothiazine-2-thiones.^a^

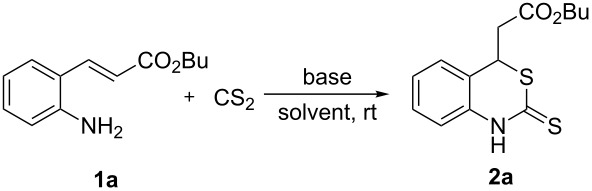			

entry	base	solvent	yield^b^ (%)

1	DBU	DMF	80
2	Et_3_N	DMF	76
3	Na_2_CO_3_	DMF	65
4	NaHCO_3_	DMF	60
5	KOH	DMF	82
6	DABCO	DMF	85
7	—	DMF	—
8^c^	DABCO	DMF	83
9^d^	DABCO	DMF	84
10^e^	DABCO	DMF	44
11^d^	DABCO	toluene	—
12^d^	DABCO	CH_2_Cl_2_	trace
13^d^	DABCO	DMSO	88
14^d^	DABCO	1,4-dioxane	45
15^d^	DABCO	CH_3_CN	50
16^d^	DABCO	THF	30
17^d,f^	DABCO	DMSO	65

^a^Reaction conditions: (*E*)-butyl 3-(2-aminophenyl)acrylate (**1a**, 0.3 mmol), CS_2_ (3 mmol, 10.0 equiv), base (0.3 mmol), rt, 2 d. ^b^Isolated yield based on **1a**. ^c^CS_2_ (1.8 mmol, 6.0 equiv). ^d^CS_2_ (1.2 mmol, 4.0 equiv). ^e^CS_2_ (0.9 mmol, 3.0 equiv). ^f^Reaction performed in DMSO at 60 °C in sealed tube.

With the preliminary optimized reaction conditions in hand, we next tested the generality of the (*E*)-3-(2-aminoaryl)acrylates (Table 2). As expected, a series of functional groups on the phenyl ring of the (*E*)-butyl 3-(2-aminoaryl)acrylates, such as methyl, chloro, fluoro, and nitro were compatible in this procedure, and the corresponding desired products **2b**–**2e** were isolated in 36–86% yields. In general, substrates with electron-donating (methyl) and weakly or moderately electron-withdrawing groups (F, Cl) showed good results in the transformation. For instance, (*E*)-butyl 3-(2-amino-5-methylphenyl)acrylate (**1b**) reacted with CS_2_ leading to the corresponding product **2b** in 75% yield (Table 2, entry 2). A slightly higher yield was obtained when (*E*)-butyl 3-(2-amino-5-fluorophenyl)acrylate (**1d**) was used as a replacement in the above reaction (86% yield, Table 2, entry 4). It is worth noting that a substrate with strongly electron-withdrawing group (nitro) gave a low yield 36% of the product **2e**. Further exploration indicated that various alkyl (methyl, ethyl, *tert*-butyl) 3-(2-aminophenyl)acrylates **1** were suitable reactants in the transformation, and the desired products **2f–2j** were obtained in moderate to good yields (Table 2, entries 6–10). When (*E*)-ethyl 3-(2-aminophenyl)acrylate (**1g**) was employed in the reaction, the corresponding product **2g** was isolated in 80% yield (Table 2, entry 7). We next examined the reaction of (*E*)-methyl 3-(2-aminophenyl)-2-methylacrylates **1k–1n** with different substituents on the phenyl ring, and the desired products **2k–2n** were isolated in 54–74% yield (Table 2, entries 11–14). Furthermore, the reaction conditions proved to be useful for (*E*)-3-(2-aminoaryl)acrylonitriles (**1o–1r**, Table 2, entries 15–18). For instance, (*E*)-3-(2-aminophenyl)acrylonitrile (**1o**) reacted with CS_2_ affording the expected product **2o** in excellent 90% yield (Table 2, entry 15). However, it was found that reactants 2-(2-aminobenzylidene)malononitrile (**1s**) and ethyl 3-(2-aminophenyl)-2-cyanoacrylate (**1t**) were not workable under the standard conditions (Table 2, entries 19 and 20).

**Table 2:** Preparation of 4-alkyl-4*H*-3,1-benzothiazine-2-thione derivatives **2**.^a^

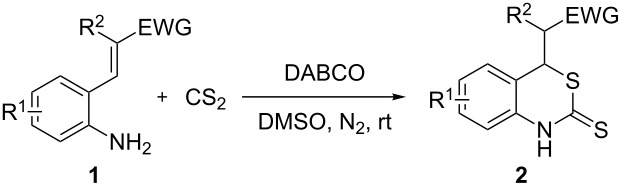			

entry	substrate **1**	product **2**	yield^b^ (%)

1	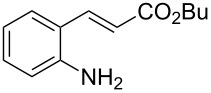 **1a**	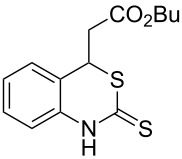 **2a**	88
2	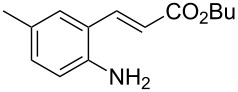 **1b**	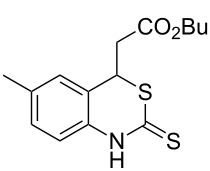 **2b**	75
3	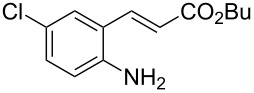 **1c**	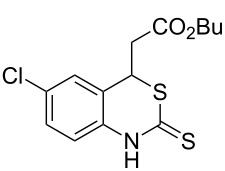 **2c**	73
4	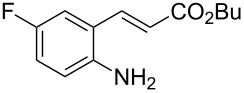 **1d**	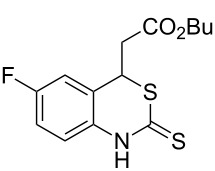 **2d**	86
5	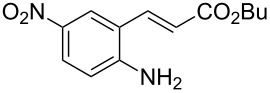 **1e**	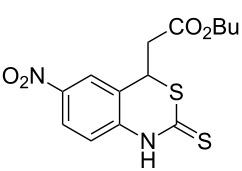 **2e**	36
6	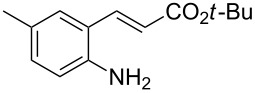 **1f**	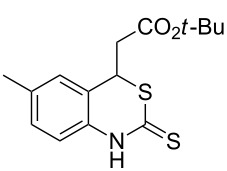 **2f**	87
7	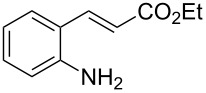 **1g**	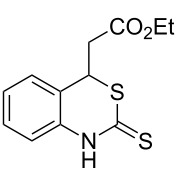 **2g**	80
8	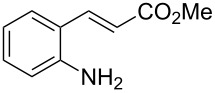 **1h**	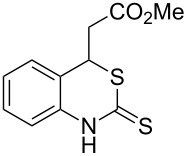 **2h**	60
9	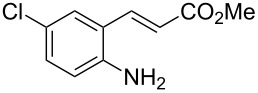 **1i**	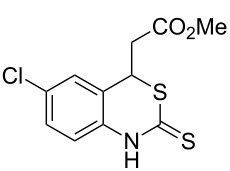 **2i**	72
10	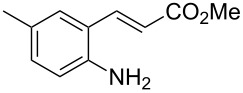 **1j**	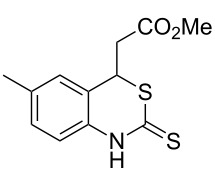 **2j**	71
11	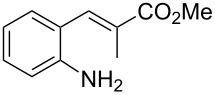 **1k**	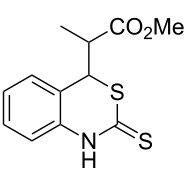 **2k**	74
12	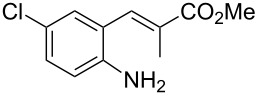 **1l**	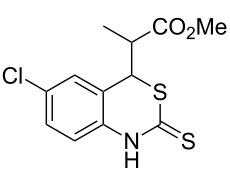 **2l**	55
13	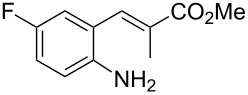 **1m**	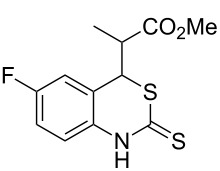 **2m**	54
14	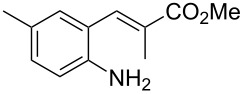 **1n**	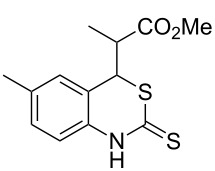 **2n**	74
15	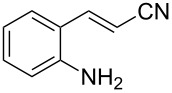 **1o**	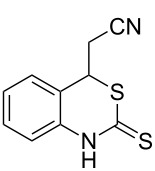 **2o**	90
16	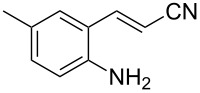 **1p**	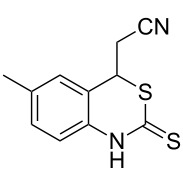 **2p**	75
17	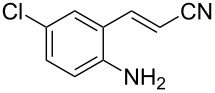 **1q**	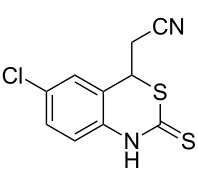 **2q**	44
18	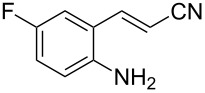 **1r**	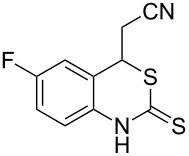 **2r**	53
19	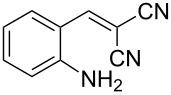 **1s**	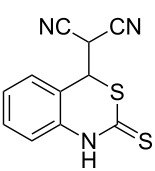 **2s**	NR
20	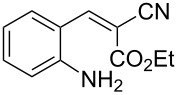 **1t**	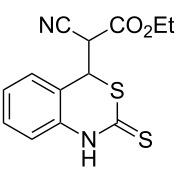 **2t**	NR

^a^Reaction conditions: substrate **1** (0.3 mmol), CS_2_ (1.2 mmol, 4.0 equiv), base (0.3 mmol, 1.0 equiv), rt, 2 d. ^b^Isolated yield based on **1**.

**Scheme 3:**
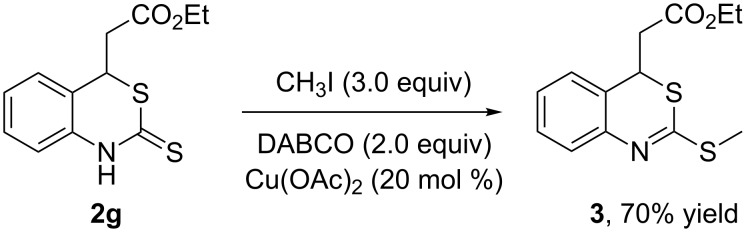
Alkylation of **2g** with iodomethane.

The 2-(2-thioxo-2,4-dihydro-1*H*-benzo[*d*][1,3]thiazin-4-yl)acetate **2** could be further elaborated by alkylation with alkyl halide. For example, compound **2g** reacted with iodomethane to afford the expected ethyl 2-(2-(methylthio)-4*H*-benzo[*d*][1,3]thiazin-4-yl)acetate (**3**) in 70% yield (Scheme 3).

## Conclusion

In summary, we have successfully developed a new simple and efficient method to construct ring-fused 4-alkyl-4*H*-3,1-benzothiazine-2-thione derivatives. In the context of this method, carbon disulfide reacted with (*E*)-3-(2-aminoaryl)acrylates or (*E*)-3-(2-aminoaryl)acrylonitriles under metal-free conditions at room temperature. The newly developed method tolerates a wide range of substrates in moderate to excellent yields and provides promise for further alkylation or arylation. Moreover, this method is advantageous over previous ones [16–17] for the easy synthesis of reactants.

## Experimental

### General

All reactions were performed in test tubes in air. Flash column chromatography was performed with silica gel (200–300 mesh). Analytical thin-layer chromatography was performed on glass plates precoated with 0.25 mm 230–400 mesh silica gel impregnated with a fluorescent indicator (254 nm). Thin-layer chromatography plates were visualized by exposure to ultraviolet light. Organic solutions were concentrated on rotary evaporators at 25–35 °C. Commercial reagents and solvents were used as received. ^1^H and ^13^C NMR spectra were recorded on a Bruker AV 400 at 400 MHz (^1^H) and 100 MHz (^13^C) at ambient temperature. Chemical shifts are reported in parts per million (ppm) on the delta scale (δ) and referenced to tetramethylsilane (0 ppm). HRMS analyses were performed in ESI mode on a Bruker mass spectrometer.

General procedure for the synthesis of 2-(2-thioxo-2,4-dihydro-1*H*-benzo[*d*][1,3]thiazin-4-yl)acetate, **2**: A mixture of 3-(2-aminoaryl)acrylate **1** (0.3 mmol), CS_2_ (1.2 mmol, 4.0 equiv, 91.2 mg) and DABCO (0.3 mmol, 1.0 equiv, 33.6 mg) was stirred in DMSO (2 mL) at room temperature. After completion of the reaction as indicated by TLC (about 2 d), the reaction was quenched by water and extracted with ethyl acetate. The organic layers were dried with anhydrous MgSO_4_, the solvent was evaporated under vacuum, and the residue was isolated by column chromatography with EtOAc/petroleum ether (1/5, v/v) as eluent to yield the desired products **2**. For details, see Supporting Information File 1.

## Supporting Information

File 1 General procedure, characterization data and copies of spectra.
