# Supplementary material for: New simple synthesis of ring-fused 4-alkyl-4H-3,1-benzothiazine-2-thiones: Direct formation from carbon disulfide and (E)-3-(2-aminoaryl)acrylates or (E)-3-(2-aminoaryl)acrylonitriles
Source: Beilstein J Org Chem. 2013 Mar 1;9:460–6. doi: 10.3762/bjoc.9.49 (PMC3596062; doi:10.3762/bjoc.9.49)
Supplement: File 1 — General procedure, characterization data and copies of spectra. [file Beilstein_J_Org_Chem-09-460-s001.pdf]

Supporting Information

for

New simple synthesis of ring-fused 4-alkyl-4*H*-3,1-benzothiazine-2-thiones: direct formation from carbon disulfide and (*E*)-3-(2-aminoaryl)acrylates or (*E*)-3-(2-aminoaryl)acrylonitriles

Qiuping Ding*, Yuqing Lin, Guangni Ding, Fumin Liao, Xiaoyan Sang and Yi-Yuan Peng*

Address: Key Laboratory of Functional Small Organic Molecules, Ministry of Education and College of Chemistry & Chemical Engineering, Jiangxi Normal University, Nanchang, Jiangxi 330022, China.

E-mail: Qiuping Ding - dqpjxnu@gmail.com; Yi-Yuan Peng - yiyuanpeng@yahoo.com

*Corresponding author

General procedure, characterization data and copies of spectra

General procedure S2

Characterization data for compounds **2** and **3** S2–S9

Copies of NMR spectra S10–S28

General procedure for the synthesis of 2-(2-thioxo-2,4-dihydro-1*H*-benzo[*d*][1,3]thiazin-4-yl)acetate, **2**:

A mixture of 3-(2-aminoaryl)acrylate **1** (0.3 mmol), CS_2 (1.2 mmol, 4.0 equiv, 91.2 mg), and DABCO (0.3 mmol, 1.0 equiv, 33.6 mg) was stirred in DMSO (2 mL) at room temperature. After completion of the reaction as indicated by TLC (about 2 d), the reaction was quenched by water and extracted with ethyl acetate. The organic layers were dried with anhydrous $MgSO_4$, the solvent was evaporated under vacuum, and the residue was isolated by column chromatography with EtOAc/petroleum ether (1/5, v/v) as eluent to yield the desired products **2**.

Butyl 2-(2-thioxo-2,4-dihydro-1*H*-benzo[*d*][1,3]thiazin-4-yl)acetate (**2a**): yellow oil (77.9 mg, 88%); R_f 0.33; ^{1}H NMR (400 MHz, $CDCl_3$) δ 0.90 (t, *J* = 7.6 Hz, 3H), 1.27-1.35 (m, 2H), 1.53-1.59 (m, 2H), 2.79 (dd, *J* = 7.2, 6.8 Hz, 1H), 2.95 (dd, *J* = 8.4, 8.0 Hz, 1H), 4.09 (t, *J* = 6.8 Hz, 2H), 4.47 (t, *J* = 7.6 Hz, 1H), 7.11 (d, *J* = 8.0 Hz, 1H), 7.18 (t, *J* = 7.2 Hz, 1H), 7.25 (d, *J* = 7.2 Hz, 1H), 7.32 (t, *J* = 8.0 Hz, 1H), 10.75 (br, 1H); ^{13}C NMR (100 MHz, $CDCl_3$) δ 13.6, 19.0, 29.7, 42.1, 42.5, 65.1, 117.5, 121.7, 126.0, 127.6, 129.2, 136.0, 169.8, 192.7; IR (KBr) ν/cm^{-1}: 3249 (m), 3163 (m), 2932 (m), 1731 (st), 1515 (st), 1487 (st), 1340 (m), 1024 (m), 757 (m); HRMS (ESI): *m/z* $[M + H]^+$ calcd for $C_{14}H_{18}NO_2S_2$: 296.0779; found: 296.0783.

Butyl 2-(6-methyl-2-thioxo-2,4-dihydro-1*H*-benzo[*d*][1,3]thiazin-4-yl)acetate (**2b**): yellow oil (69.5 mg, 75%); R_f 0.39; ^{1}H NMR (400 MHz, $CDCl_3$) δ 0.92 (t, *J* = 7.6 Hz, 3H), 1.24-1.35 (m, 2H), 1.53-1.60 (m, 2H), 2.33 (s, 3H), 2.76 (dd, *J* = 7.2, 6.8 Hz, 1H), 2.92 (dd, *J* = 8.4, 8.0 Hz, 1H), 4.09 (t, *J* = 6.8 Hz, 2H), 4.42 (t, *J* = 7.6 Hz, 1H), 6.97 (d, *J* = 8.0 Hz, 1H), 7.05 (s, 1H), 7.12 (d, *J* = 7.6 Hz, 1H), 10.45 (br, 1H); ^{13}C NMR (100 MHz, $CDCl_3$) δ 13.7, 19.0, 20.9, 30.5, 42.2,

42.6, 65.1, 117.3, 121.6, 128.0, 129.7, 133.9, 136.0, 169.8, 192.2; IR (KBr) ν/cm^{-1}: 3244 (m), 3159 (m), 2926 (st), 1731 (st), 1500 (st), 1337 (m), 1028 (m); HRMS (ESI): *m/z* $[M + H]^+$ calcd for $C_{15}H_{20}NO_2S_2$: 310.0935; found: 310.0938.

Butyl 2-(6-chloro-2-thioxo-2,4-dihydro-1*H*-benzo[*d*][1,3]thiazin-4-yl)acetate (**2c**): yellow oil (72.0 mg, 73%); R_f 0.39; ^{1}H NMR (400 MHz, $CDCl_3$) δ 0.91 (t, *J* = 7.6 Hz, 3H), 1.25-1.35 (m, 2H), 1.53-1.60 (m, 2H), 2.80 (dd, *J* = 7.2, 7.2 Hz, 1H), 2.95 (dd, *J* = 8.0, 8.0 Hz, 1H), 4.09-4.13 (m, 2H), 4.44 (t, *J* = 7.6 Hz, 1H), 7.09 (d, *J* = 8.4 Hz, 1H), 7.26-7.30 (m, 2H), 10.92 (br, 1H); ^{13}C NMR (100 MHz, $CDCl_3$) δ 13.7, 19.0, 30.5, 41.8, 42.3, 65.3, 118.7, 123.3, 127.6, 129.2, 130.9, 134.7, 169.6, 192.4; IR (KBr) ν/ cm^{-1}: 3239 (m), 3161 (m), 2959 (st), 1731 (st), 1486 (st), 1325 (m), 1031 (m); HRMS (ESI): *m/z* $[M + H]^+$ calcd for $C_{14}H_{17}ClNO_2S_2$: 330.0389; found: 330.0392.

Butyl 2-(6-fluoro-2-thioxo-2,4-dihydro-1*H*-benzo[*d*][1,3]thiazin-4-yl)acetate (**2d**): yellow oil (80.7 mg, 86%); R_f 0.36; ^{1}H NMR (400 MHz, $CDCl_3$) δ 0.91 (t, *J* = 7.6 Hz, 3H), 1.26-1.35 (m, 2H), 1.55-1.60 (m, 2H), 2.81 (dd, *J* = 7.6, 7.6 Hz, 1H), 2.95 (dd, *J* = 8.0, 8.0 Hz, 1H), 4.11 (t, *J* = 6.8 Hz, 1H), 4.45 (t, *J* = 7.6 Hz, 1H), 6.99-7.07 (m, 2H), 7.15 (dd, *J* = 3.2, 8.0 Hz, 1H), 11.07 (br, 1H); ^{13}C NMR (100 MHz, $CDCl_3$) δ 13.7, 19.0, 30.5, 41.8, 42.1, 65.3, 114.6 (d, $^2J_{C\text{-}F}$ = 23.0 Hz), 116.2 (d, $^2J_{C\text{-}F}$ = 23.0 Hz), 119.1 (d, $^2J_{C\text{-}F}$ = 8.0 Hz), 123.8 (d, $^2J_{C\text{-}F}$ = 8.0 Hz), 132.5, 159.8 (d, $^1J_{C\text{-}F}$ = 247.0 Hz), 169.7, 192.0; IR (KBr) ν/ cm^{-1}: 3165 (m), 2960 (st), 1731 (st), 1494 (st), 1344 (m), 1027 (m); HRMS (ESI): *m/z* $[M + H]^+$ calcd for $C_{14}H_{17}FNO_2S_2$: 314.0685; found: 314.0690.

Butyl 2-(6-nitro-2-thioxo-2,4-dihydro-1*H*-benzo[*d*][1,3]thiazin-4-yl)acetate (**2e**): yellow solid (36.7 mg, 36%); R_f 0.33; mp 98-99 °C; ^{1}H NMR (400 MHz, $CDCl_3$) δ 0.92 (t, *J* = 7.6 Hz, 3H), 1.27-1.36 (m, 2H), 1.56-1.62 (m, 2H), 2.86 (dd, *J* = 7.6, 7.2 Hz, 1H), 2.95 (dd, *J* = 7.6, 7.6 Hz, 1H), 4.10-4.13 (m, 2H), 4.60 (t, *J* = 7.6 Hz, 1H), 7.21 (d, *J* = 9.6 Hz, 1H), 8.22-8.24 (m, 2H), 10.56 (br, 1H); ^{13}C NMR (100 MHz, $CDCl_3$) δ 13.2, 18.5, 29.9, 41.2, 41.5, 65.0, 117.2, 122.0, 123.4, 124.5, 139.9, 143.8, 168.7, 193.1; IR (KBr) ν/ cm^{-1}: 3203 (m), 2959 (st), 1728 (st), 1337 (m), 1290 (m); HRMS (ESI): *m/z* $[M + H]^+$ calcd for $C_{14}H_{17}N_2O_4S_2$: 341.0630; found: 341.0627.

tert-Butyl 2-(6-methyl-2-thioxo-2,4-dihydro-1*H*-benzo[*d*][1,3]thiazin-4-yl)acetate (**2f**): yellow solid (80.6 mg, 87%); R_f 0.15; mp 115-116 °C; ^{1}H NMR (400 MHz, $CDCl_3$) δ 1.43 (s, 9H), 2.30 (s, 3H), 2.67-2.73 (m, 1H), 2.79-2.84 (m, 1H), 4.39 (t, *J* = 7.6 Hz, 1H), 4.42 (t, *J* = 7.6 Hz, 1H), 7.03 (s, 1H), 7.05 (d, *J* = 8.0 Hz, 1H), 7.10 (d, *J* = 8.0 Hz, 1H), 11.08 (br, 1H); ^{13}C NMR (100 MHz, $CDCl_3$) δ20.9, 28.0, 42.3, 42.8, 81.8, 117.4, 121.6, 128.0, 129.6, 133.9, 135.7, 169.0, 191.7; IR (KBr) ν/ cm^{-1}: 3440 (m), 3149 (m), 2977 (st), 1603 (m), 1497 (st), 1348 (st), 1025 (st); HRMS (ESI): *m/z* $[M + H]^+$ calcd for $C_{15}H_{20}NO_2S_2$: 310.0935; found: 310.0932.

Ethyl 2-(2-thioxo-2,4-dihydro-1*H*-benzo[*d*][1,3]thiazin-4-yl)acetate (**2g**): yellow solid (64.1 mg, 80%); R_f 0.27; mp 96-97 °C; ^{1}H NMR (400 MHz, $CDCl_3$) δ 1.11 (t, *J* = 7.6 Hz, 3H), 2.70 (dd, *J* = 8.0, 8.0 Hz, 1H), 2.84 (dd, *J* = 8.0, 8.0 Hz, 1H), 4.05 (t, *J* = 8.0 Hz, 2H), 4.42 (t, *J* = 8.0 Hz, 1H), 7.05-7.20 (m, 4H), 10.07 (br, 1H); ^{13}C NMR (100 MHz, $CDCl_3$) δ 14.5, 42.4, 42.9, 65.6, 117.9, 121.9, 126.3, 127.9, 129.5, 136.4, 170.2, 192.6; IR (KBr) ν/ cm^{-1}: 3431 (m), 2980 (st), 1727 (st), 1486 (m), 1347 (m), 1021 (m); HRMS (ESI): *m/z* $[M + H]^+$ calcd for $C_{12}H_{14}NO_2S_2$: 268.0466; found: 268.0470.

Methyl 2-(2-thioxo-2,4-dihydro-1*H*-benzo[*d*][1,3]thiazin-4-yl)acetate (**2h**): yellow solid (45.5 mg, 60%); R_f 0.27; mp 119-120 ^{o}C; ^{1}H NMR (400 MHz, $CDCl_3$) δ 2.80 (dd, *J* = 6.4, 6.8 Hz, 1H), 2.96 (dd, *J* = 8.4, 8.4 Hz, 1H), 3.68 (s, 3H), 4.48 (t, *J* = 8.0 Hz, 1H), 7.14 (d, *J* = 7.6 Hz, 1H), 7.18 (t, *J* = 7.6 Hz, 1H), 7.25 (d, *J* = 7.2 Hz, 1H), 7.32 (t, *J* = 7.6 Hz, 1H), 10.89 (br, 1H); ^{13}C NMR (100 MHz, $CDCl_3$) δ42.0, 42.3, 52.2, 117.6, 121.6, 126.0, 127.6, 129.2, 136.1, 170.2, 192.5; IR (KBr) ν/cm^{-1}: 3453 (w), 3160 (m), 2952 (st), 1731 (st), 1486 (st), 1245 (m), 1029 (m); HRMS (ESI): *m/z* $[M + H]^+$ calcd for $C_{11}H_{12}NO_2S_2$: 254.0309; found: 254.0302.

Methyl 2-(6-chloro-2-thioxo-2,4-dihydro-1*H*-benzo[*d*][1,3]thiazin-4-yl)acetate (**2i**): yellow solid (62.0 mg, 72%); R_f 0.27; mp 120-121 ^{o}C; ^{1}H NMR (400 MHz, $CDCl_3$) δ2.80 (dd, *J* = 8.0, 8.0 Hz, 1H), 2.96 (dd, *J* = 8.0, 8.0 Hz, 1H), 3.69 (s, 3H), 4.44 (t, *J* = 8.0 Hz, 1H), 7.10 (d, *J* = 8.0 Hz, 1H), 7.25 (s, 1H), 7.27 (d, *J* = 8.0 Hz, 1H), 11.00 (br, 1H); ^{13}C NMR (100 MHz, $CDCl_3$) δ42.1, 42.4, 52.6, 119.1, 123.7, 127.9, 129.6, 131.2, 135.1, 170.3, 192.6; IR (KBr) ν/cm^{-1}: 3434 (m), 3145 (m), 2967 (st), 1727 (st), 1517 (st), 1484 (m), 1347 (m), 1034 (m); HRMS (ESI): *m/z* $[M + H]^+$ calcd for $C_{11}H_{11}ClNO_2S_2$: 287.9920; found: 287.9925.

Methyl 2-(6-methyl-2-thioxo-2,4-dihydro-1*H*-benzo[*d*][1,3]thiazin-4-yl)acetate (**2j**): yellow solid (56.9 mg, 71%); R_f 0.24; mp 83-84 ^{o}C; ^{1}H NMR (400 MHz, $CDCl_3$) δ2.23 (s, 3H), 2.69 (dd, *J* = 6.4, 6.4 Hz, 1H), 2.85 (dd, *J* = 8.4, 8.4 Hz, 1H), 3.60 (s, 3H), 4.33 (t, *J* = 8.0 Hz, 1H), 6.95 (d, *J* = 8.4 Hz, 1H), 6.96 (s, 1H), 7.02 (d, *J* = 8.4 Hz, 1H), 10.86 (br, 1H); ^{13}C NMR (100 MHz, $CDCl_3$) δ20.9, 42.1, 42.4, 52.1, 117.5, 121.5, 127.9, 129.7, 133.9, 136.0, 170.3, 191.6; IR (KBr) ν/cm^{-1}: 3164 (m), 2922 (st), 1731 (st), 1504 (st), 1025 (m); HRMS (ESI): *m/z* $[M + H]^+$ calcd for $C_{12}H_{14}NO_2S_2$: 268.0466; found: 268.0468.

Methyl 2-(2-thioxo-2,4-dihydro-1*H*-benzo[*d*][1,3]thiazin-4-yl)propanoate (**2k**): yellow solid (59.3 mg, 74%); R_f 0.32; mp 105-106 °C; ^{1}H NMR (400 MHz, $CDCl_3$) δ 1.02 (d, *J* = 6.8 Hz, 0.65H), 1.33 (d, *J* = 6.8 Hz, 2.35H), 2.83-2.87 (m, 1H), 3.55 (s, 2.35H), 3.76 (s, 0.65H), 4.13 (d, *J* = 7.2 Hz, 0.22H), 4.30 (d, *J* = 8.0 Hz, 0.78H), 7.05-7.10 (m, 1H), 7.12-7.18 (m, 2H), 7.30-7.34 (m, 1H), 10.45 (br, 1H); ^{13}C NMR (100 MHz, $CDCl_3$) δ13.5, 46.9, 48.9, 52.1, 117.3, 120.6, 125.7, 128.2, 129.2, 136.3, 173.4, 192.6; IR (KBr) ν/cm^{-1}: 3459 (w), 3162 (m), 2985 (st), 1733 (st), 1487 (st), 1347 (m), 1021 (m); HRMS (ESI): *m/z* $[M + H]^+$ calcd for $C_{12}H_{14}NO_2S_2$: 268.0466; found: 268.0461.

Methyl 2-(6-chloro-2-thioxo-2,4-dihydro-1*H*-benzo[*d*][1,3]thiazin-4-yl)propanoate (**2l**): yellow solid (49.7 mg, 55%); R_f 0.48; mp 99-100 °C; ^{1}H NMR (400 MHz, $CDCl_3$) δ 1.06 (d, *J* = 6.8 Hz, 0.6H), 1.33 (d, *J* = 6.8 Hz, 2.4H), 2.83-2.91 (m, 1H), 3.61 (s, 2.4H), 3.77 (s, 0.6H), 4.15 (d, *J* = 7.6 Hz, 0.2H), 4.27 (d, *J* = 7.6 Hz, 0.8H), 7.03 (d, *J* = 8.4 Hz, 1H), 7.17 (s, 1H), 7.29 (d, *J* = 8.8 Hz, 1H), 10.65 (br, 1H); ^{13}C NMR (100 MHz, $CDCl_3$) δ13.4, 46.8, 48.5, 52.2, 118.5, 122.3, 128.1, 129.2, 130.6, 134.9, 173.2, 192.3; IR (KBr) ν/cm^{-1}: 3441 (m), 3243 (m), 2973 (st), 1732 (st), 1486 (st), 1333 (m), 1026 (m); HRMS (ESI): *m/z* $[M + H]^+$ calcd for $C_{12}H_{13}ClNO_2S_2$: 302.0076; found: 302.0081.

Methyl 2-(6-fluoro-2-thioxo-2,4-dihydro-1*H*-benzo[*d*][1,3]thiazin-4-yl)propanoate (**2m**): yellow solid (46.2 mg, 54%); R_f 0.41; mp 142-143 °C; ^{1}H NMR (400 MHz, $CDCl_3$) δ 0.99 (d, *J* = 7.2 Hz, 1.5H), 1.26 (d, *J* = 7.2 Hz, 1.5H), 2.84-2.95 (m, 1H), 3.61 (s, 1.5H), 3.77 (m, 1.5H), 4.18 (d, *J* = 9.6 Hz, 0.5H), 4.27 (d, *J* = 7.6 Hz, 0.5H), 6.92 (d, *J* = 8.0 Hz, 1H), 7.02-7.15 (m, 2H), 10.83 (br, 0.5H), 10.90 (br, 0.5H); ^{13}C NMR (100 MHz, $CDCl_3$) δ 13.6, 46.6, 47.8, 52.4, 115.1 (d, $^2J_{C-F}$ = 23.0 Hz), 115.8 (d, $^2J_{C-F}$ = 23.0 Hz), 116.4 (d, $^2J_{C-F}$ = 8.0 Hz), 121.8 (d, $^2J_{C-F}$ = 8.0 Hz),

133.0, 159.5 (d, $^{1}J_{C\text{-}F}$ = 246.0 Hz), 173.3, 192.5; 15.5, 46.1, 48.6, 52.2, 115.1 (d, $^{2}J_{C\text{-}F}$ = 23.0 Hz), 115.8 (d, $^{2}J_{C\text{-}F}$ = 23.0 Hz), 116.2 (d, $^{2}J_{C\text{-}F}$ = 8.0 Hz), 122.7 (d, $^{2}J_{C\text{-}F}$ = 8.0 Hz), 132.8, 159.7 (d, $^{1}J_{C\text{-}F}$ = 246.0 Hz), 173.9, 191.7; IR (KBr) ν/cm^{-1}: 3161 (w), 2986 (w), 1731 (st), 1494 (st), 1337 (m), 1011 (m); HRMS (ESI): *m/z* $[M + H]^+$ calcd for $C_{12}H_{13}FNO_2S_2$: 286.0372; found: 286.0375.

Methyl 2-(6-methyl-2-thioxo-2,4-dihydro-1*H*-benzo[*d*][1,3]thiazin-4-yl)propanoate (**2n**): yellow solid (62.4 mg, 74%); R_f 0.41; mp 151-152 °C; ^{1}H NMR (400 MHz, $CDCl_3$) δ 1.02 (d, *J* = 7.2 Hz, 1.2H), 1.31 (d, *J* = 7.2 Hz, 1.8H), 2.32 (s, 1.8H), 2.34 (s, 1.2H), 2.83-2.91 (m, 1H), 3.57 (s, 1.8H), 3.76 (s, 1.2H), 4.14 (d, *J* = 7.6 Hz, 0.4H), 4.28 (d, *J* = 7.6 Hz, 0.6H), 6.92-7.00 (m, 2H), 7.10-7.15 (m, 1H), 10.41 (br, 0.6H), 10.49 (br, 0.4H); ^{13}C NMR (100 MHz, $CDCl_3$) δ13.2, 20.9, 47.1, 48.9, 52.0, 117.3, 120.4, 128.6, 129.8, 134.1, 135.8, 173.4, 190.9; 15.6, 21.0, 46.3, 48.1, 52.2, 117.2, 119.6, 129.4, 129.9, 134.2, 135.5, 174.2, 192.2; IR (KBr) ν/cm^{-1}: 3450 (w), 3157 (m), 2977 (st), 1731 (st), 1455(st), 1024 (m); HRMS (ESI): *m/z* $[M + H]^+$ calcd for $C_{13}H_{16}NO_2S_2$: 282.0622; found: 282.0618.

2-(2-Thioxo-2,4-dihydro-1*H*-benzo[*d*][1,3]thiazin-4-yl)acetonitrile (**2o**): yellow solid (59.4 mg, 90%); R_f 0.16; mp 174-175 °C; ^{1}H NMR (400 MHz, DMSO-d_6) δ 3.05 (t, *J* = 7.6 Hz, 2H), 4.64 (d, *J* = 5.6 Hz, 1H), 7.23 (t, *J* = 7.6 Hz, 1H), 7.31 (d, *J* = 7.6 Hz, 1H), 7.36-7.40 (m, 2H), 12.66 (br, 1H); ^{13}C NMR (100 MHz, DMSO-d_6) δ26.6, 40.6, 117.4, 117.8, 120.1, 125.2, 128.2, 129.3, 136.3, 189.3; IR (KBr) ν/cm^{-1}: 3441 (m), 3156 (m), 2910 (st), 2247 (w), 1609 (m), 1525 (st), 1492 (st), 1363 (m), 1014 (m); HRMS (ESI): *m/z* $[M + H]^+$ calcd for $C_{10}H_9N_2S_2$: 221.0207; found: 221.0205.

2-(6-Methyl-2-thioxo-2,4-dihydro-1*H*-benzo[*d*][1,3]thiazin-4-yl)acetonitrile (**2p**): yellow solid (52.6 mg, 75%); R_f 0.18; mp 201-202 °C; ^{1}H NMR (400 MHz, DMSO-d_6) δ 2.29 (s, 3H), 3.05 (t, *J* = 6.2 Hz, 2H), 4.61 (t, *J* = 6.4 Hz, 1H), 7.19 (d, *J* = 6.8 Hz, 3H), 12.71 (s, 1H); ^{13}C NMR (100 MHz, DMSO-d_6) δ 21.7, 28.0, 41.8, 118.8, 119.0, 121.0, 129.5, 131.0, 135.4, 135.9, 189.8; IR (KBr) ν/cm^{-1}: 3148 (m), 2957 (m), 2924 (m), 2250 (w), 1503 (st), 1027 (m); HRMS (ESI): *m/z* $[M + H]^+$ calcd for $C_{11}H_{11}N_2S_2$: 235.0364; found: 235.0362.

2-(6-Chloro-2-thioxo-2,4-dihydro-1*H*-benzo[*d*][1,3]thiazin-4-yl)acetonitrile (**2q**): yellow solid (33.5 mg, 44%); R_f 0.21; mp 226-227 °C; ^{1}HNMR (400 MHz, DMSO-d_6) δ 3.13 (s, 2H), 4.70 (s, 1H), 7.32 (d, *J* = 8.0 Hz, 1H), 7.47-7.52 (m, 2H), 12.87 (s, 1H); ^{13}C NMR (100 MHz, DMSO-d_6) δ 26.5, 40.2, 117.5, 119.5, 121.8, 127.8, 128.7, 129.3, 135.3, 189.3; IR (KBr) ν/cm^{-1}: 3438 (m), 3144 (m), 2962 (st), 2251 (w), 1508 (st), 1483 (st), 1341 (st), 1029 (st); HRMS (ESI): *m/z* $[M + H]^+$ calcd for $C_{10}H_8ClN_2S_2$: 254.9817; found: 254.9822.

2-(6-Fluoro-2-thioxo-2,4-dihydro-1*H*-benzo[*d*][1,3]thiazin-4-yl)acetonitrile (**2r**): yellow solid (37.8 mg, 53%); R_f 0.18; mp 210-212 °C; ^{1}HNMR (400 MHz, DMSO-d_6) δ 2.90-3.00 (m, 2H), 4.50 (t, 1H, *J* = 6.6 Hz, 1H), 7.08-7.19 (m, 3H), 12.66 (s, 1H); ^{13}C NMR (100 MHz, DMSO-d_6) δ 26.3, 40.3, 114.7 (d, $^2J_{C\text{-}F}$ = 24.0 Hz), 116.3 (d, $^2J_{C\text{-}F}$ = 23.0 Hz), 117.6, 119.7 (d, $^3J_{C\text{-}F}$ = 9.0 Hz), 122.0 (d, $^3J_{C\text{-}F}$ = 8.0 Hz), 133.1, 158.6 (d, $^1J_{C\text{-}F}$ = 242.0 Hz), 188.8; IR (KBr) ν/cm^{-1}: 3455 (m), 3158 (m), 2977 (st), 2250 (w), 1698 (st), 1521 (st), 1493 (st), 1351 (m), 1028 (m); HRMS (ESI): *m/z* $[M + H]^+$ calcd for $C_{10}H_8FN_2S_2$: 239.0113; found: 239.0118.

Ethyl 2-(2-(methylthio)-4*H*-benzo[*d*][1,3]thiazin-4-yl)acetate (**3**): yellow oil (59.0 mg, 70%); R_f 0.63; ^{1}H NMR (400 MHz, $CDCl_3$) δ 1.22 (t, J = 7.2 Hz, 3H), 2.60-2.66 (m, 3H), 2.69 (d, J = 8.4 Hz, 1H) 4.09-4.15 (m, 2H), 4.45 (t, J = 7.0 Hz, 1H), 7.15-7.22 (m, 2H), 7.31-7.34 (m, 2H); ^{13}C NMR (100 MHz, $CDCl_3$) δ 14.2, 14.4, 40.1, 41.4, 60.9, 123.0, 126.2, 126.7, 126.8, 128.8, 142.6, 158.9, 170.1; IR (KBr) ν/cm^{-1}: 3030 (m), 2980 (m), 1731 (st), 1537 (st), 1392 (m); HRMS (ESI): *m/z* $[M + H]^+$ calcd for $C_{13}H_{16}NO_2S_2$: 282.0622; found: 282.0618.

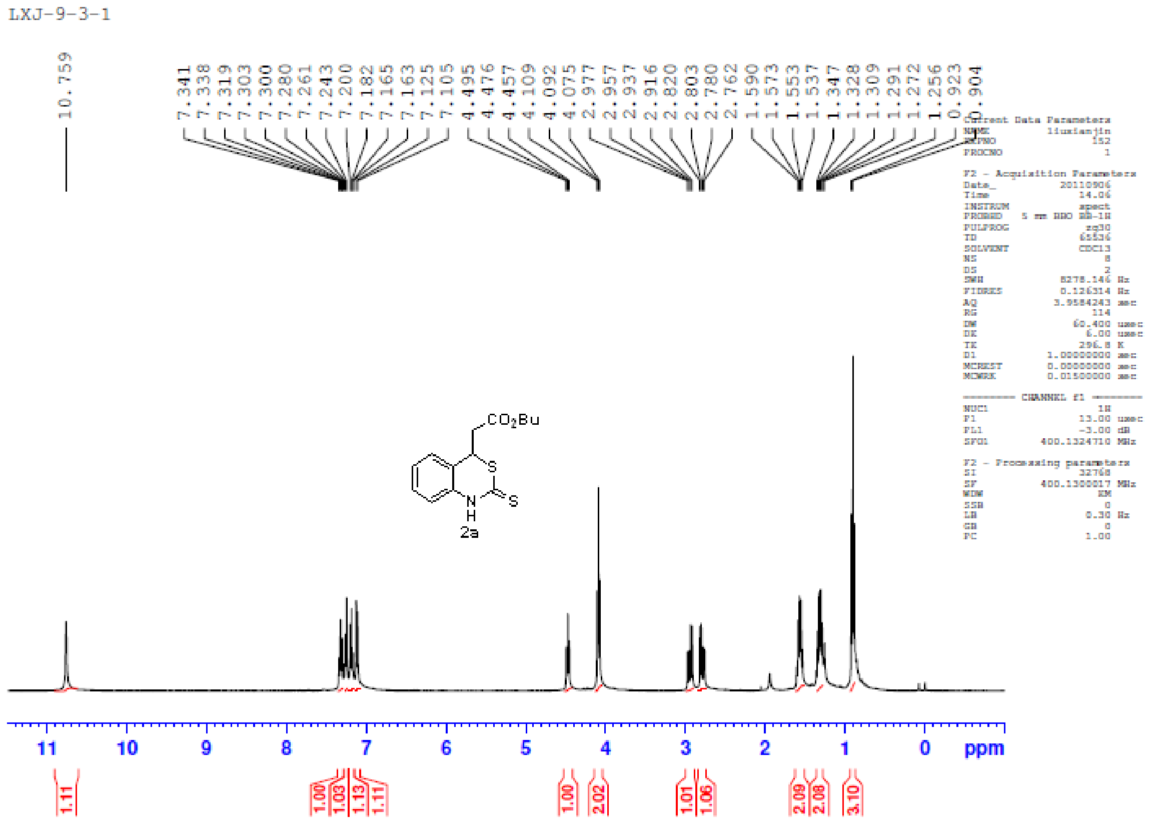
LXJ-9-3-1
10.759
7.341 7.338 7.319 7.303 7.300 7.280 7.261 7.243 7.200 7.182 7.165 7.163 7.125 7.105 4.495 4.476 4.457 4.109 4.092 4.075 2.977 2.957 2.937 2.916 2.820 2.803 2.780 2.762 1.590 1.573 1.553 1.537 1.347 1.328 1.309 1.291 1.272 1.256 0.923 0.904
CO2Bu
S
S
N
H
2a
11 10 9 8 7 6 5 4 3 2 1 0 ppm
1.11 1.00 1.03 1.13 1.11 1.00 2.02 1.01 1.06 2.09 2.08 3.10

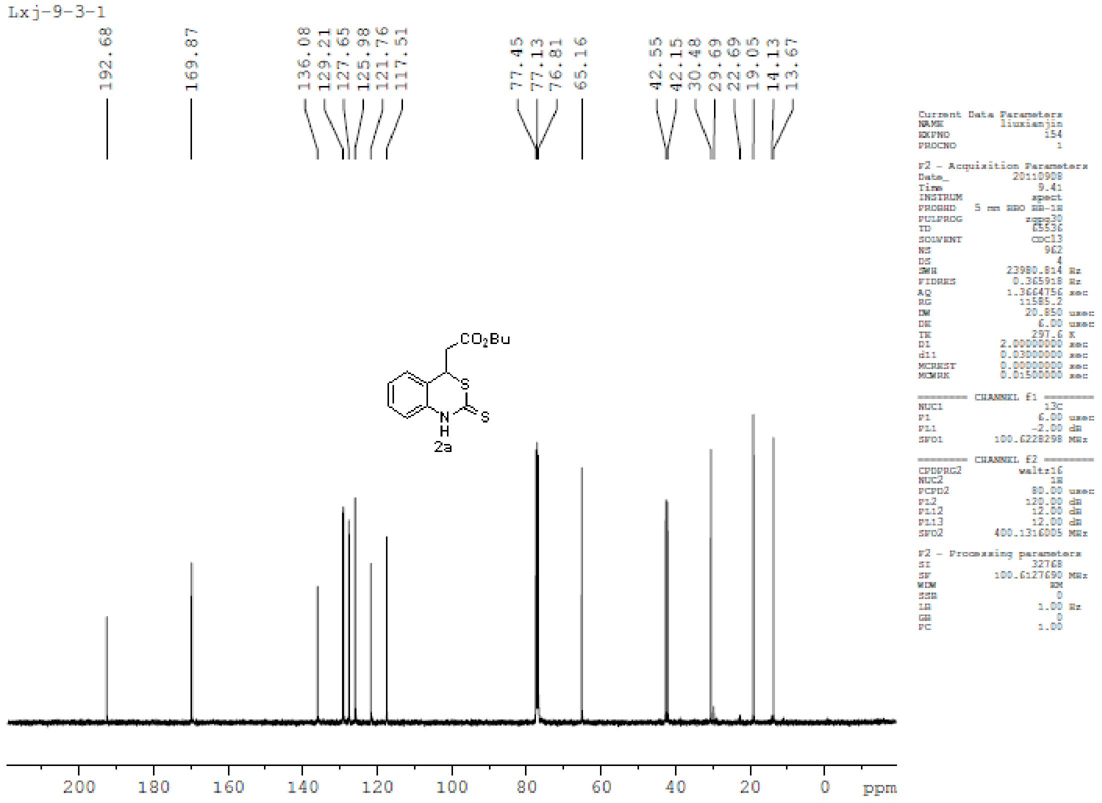
Lxj-9-3-1
192.68
169.87
136.08 129.21 127.65 125.98 121.76 117.51
77.45 77.13 76.81 65.16
42.55 42.15 30.48 29.69 22.69 19.05 14.13 13.67
CO2Bu
S
S
N
H
2a
200 180 160 140 120 100 80 60 40 20 0 ppm

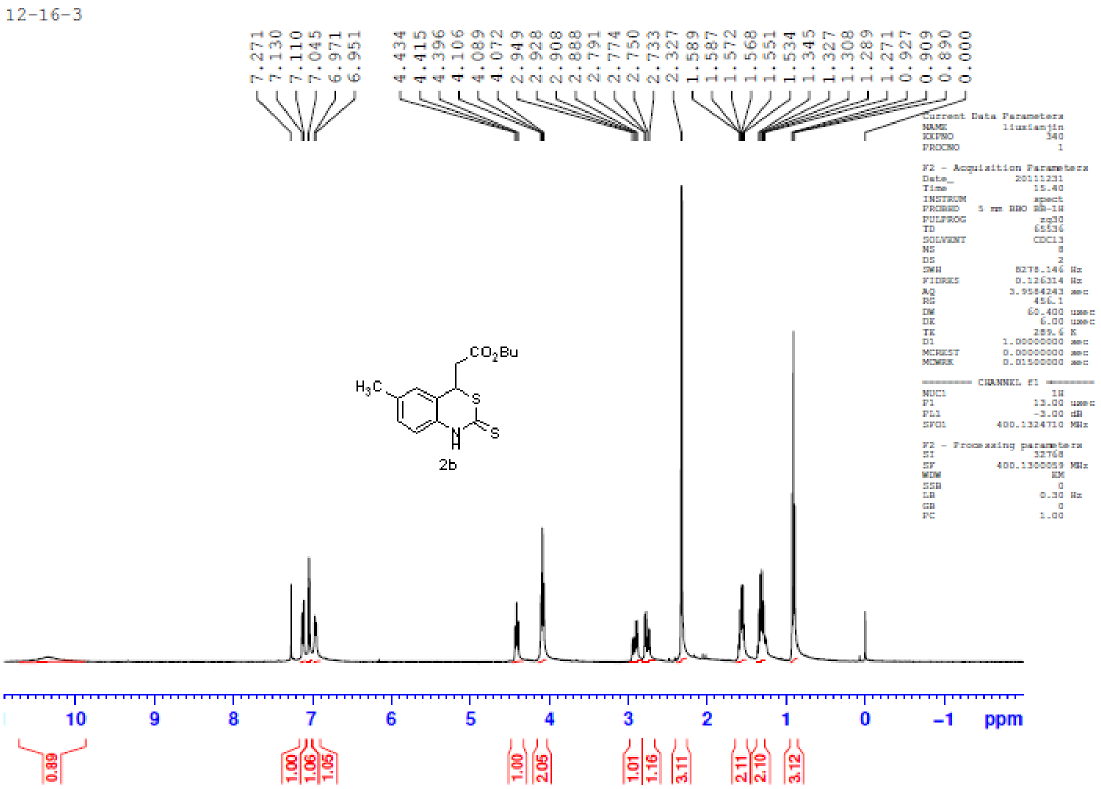
12-16-3
7.271
7.130
7.110
7.045
6.971
6.951
4.434
4.415
4.396
4.106
4.089
4.072
2.949
2.928
2.908
2.888
2.791
2.774
2.750
2.733
2.327
1.589
1.587
1.572
1.568
1.551
1.534
1.345
1.327
1.308
1.289
1.271
0.927
0.909
0.890
0.000
CO2Bu
H3C
S
S
N
H
2b
ppm
0.89
1.00
1.06
1.05
1.00
2.05
1.01
1.16
3.11
2.11
2.10
3.12

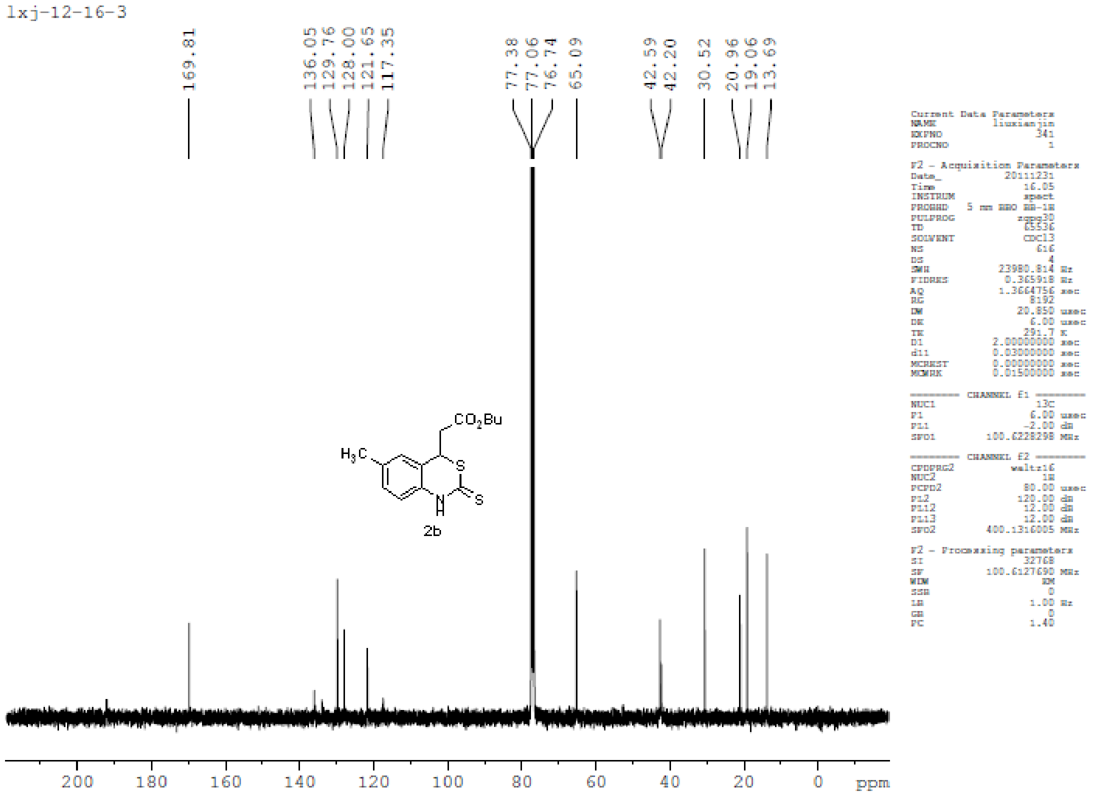
lxj-12-16-3
169.81
136.05
129.76
128.00
121.65
117.35
77.38
77.06
76.74
65.09
42.59
42.20
30.52
20.96
19.06
13.69
CO2Bu
H3C
S
S
N
H
2b
ppm

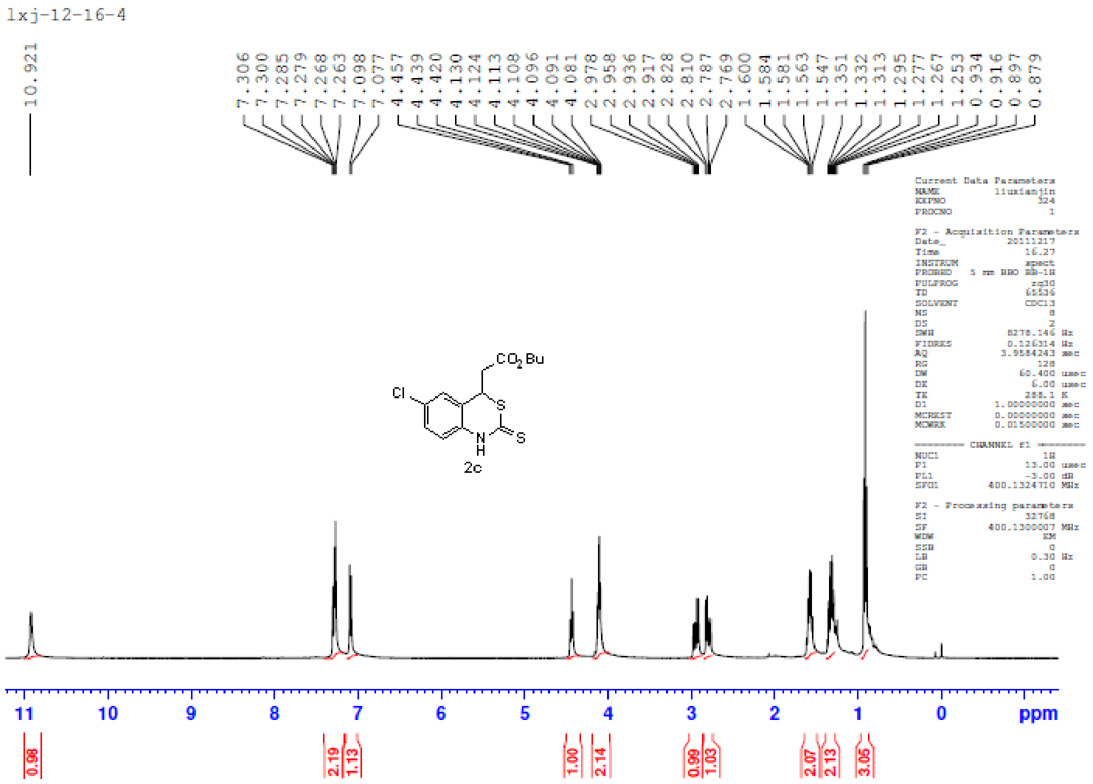

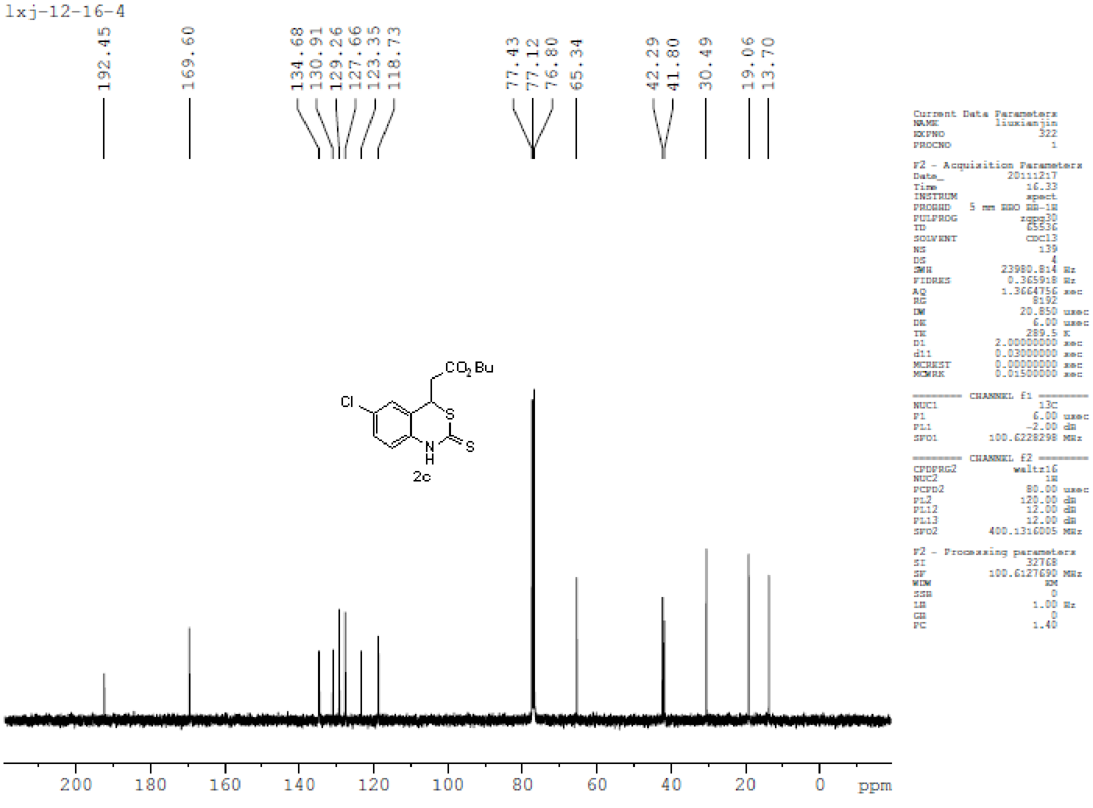

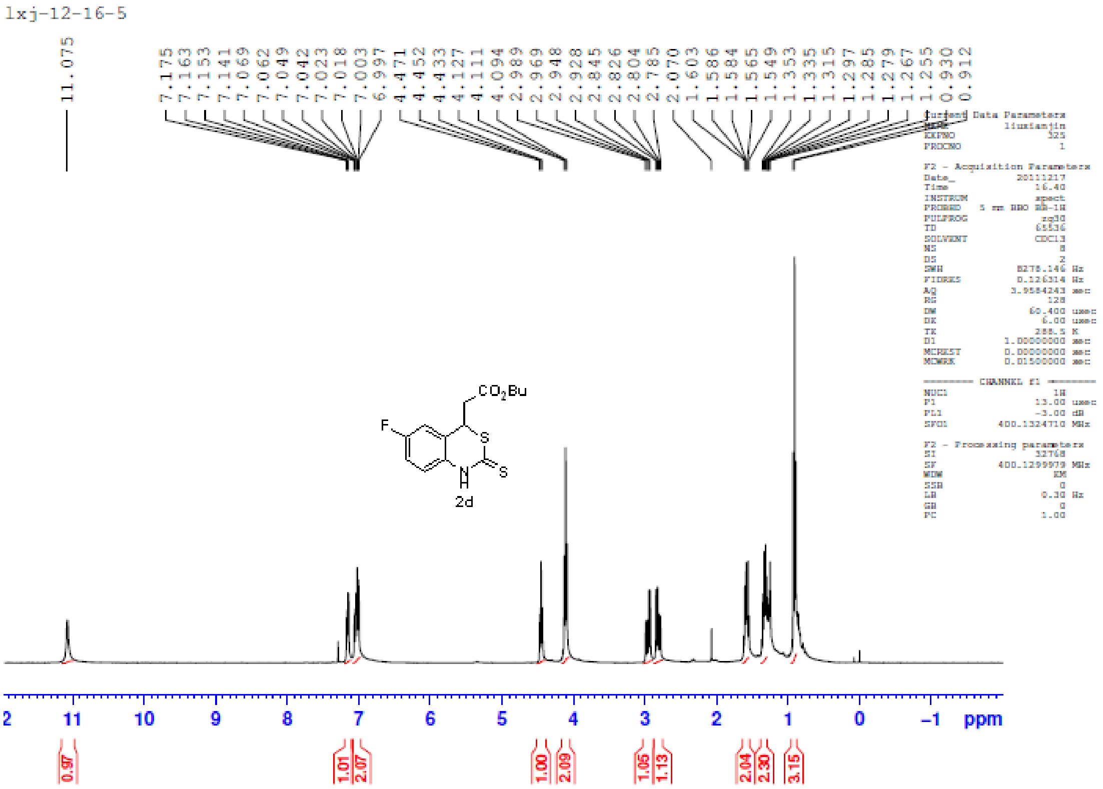
lxj-12-16-5
11.075
7.175 7.163 7.153 7.141 7.069 7.062 7.049 7.042 7.023 7.018 7.003 6.997
4.471 4.452 4.433 4.127 4.111 4.094
2.989 2.969 2.948 2.928 2.845 2.826 2.804 2.785
2.070
1.603 1.586 1.584 1.565 1.549 1.353 1.335 1.315 1.297 1.285 1.279 1.267 1.255
0.930 0.912
F
CO_2Bu
S
S
N
H
2d
0.97 1.01 2.07 1.00 2.09 1.05 1.13 2.04 2.30 3.15
2 11 10 9 8 7 6 5 4 3 2 1 0 −1 ppm

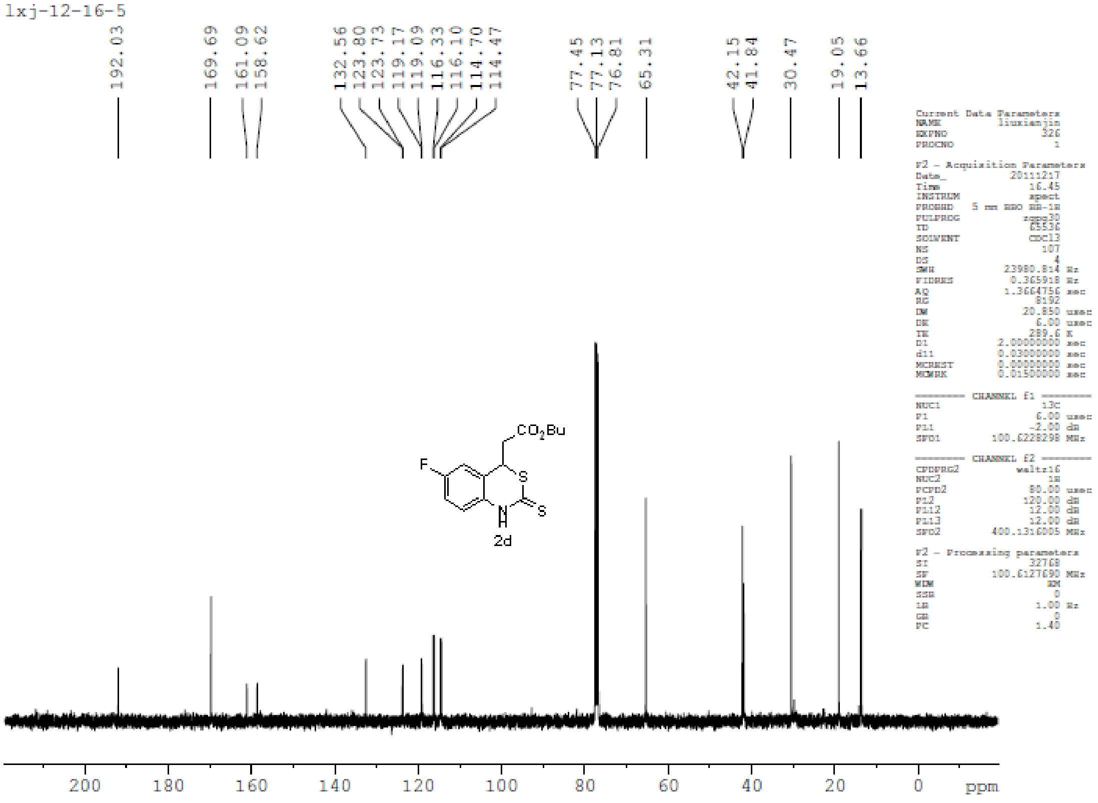
lxj-12-16-5
192.03
169.69 161.09 158.62
132.56 123.80 123.73 119.17 119.09 116.33 116.10 114.70 114.47
77.45 77.13 76.81
65.31
42.15 41.84
30.47
19.05 13.66
F
CO_2Bu
S
S
N
H
2d
200 180 160 140 120 100 80 60 40 20 0 ppm

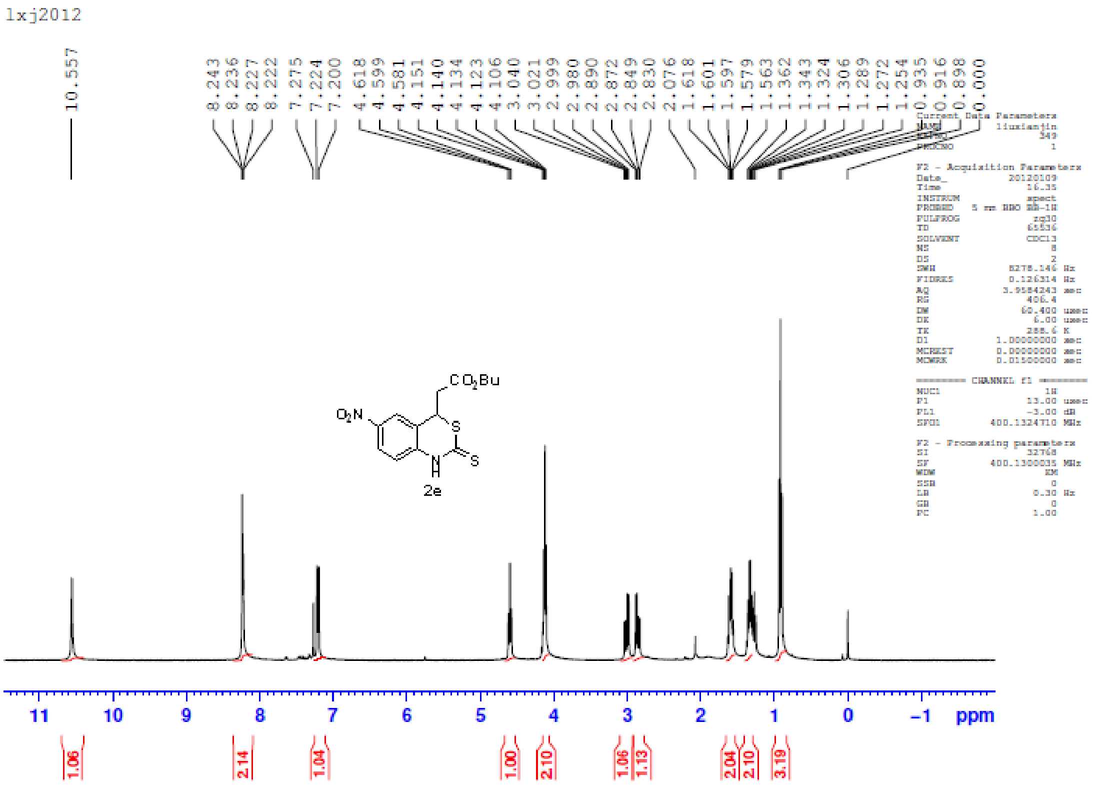

lxj2012
2e
11
10
9
8
7
6
5
4
3
2
1
0
-1
ppm

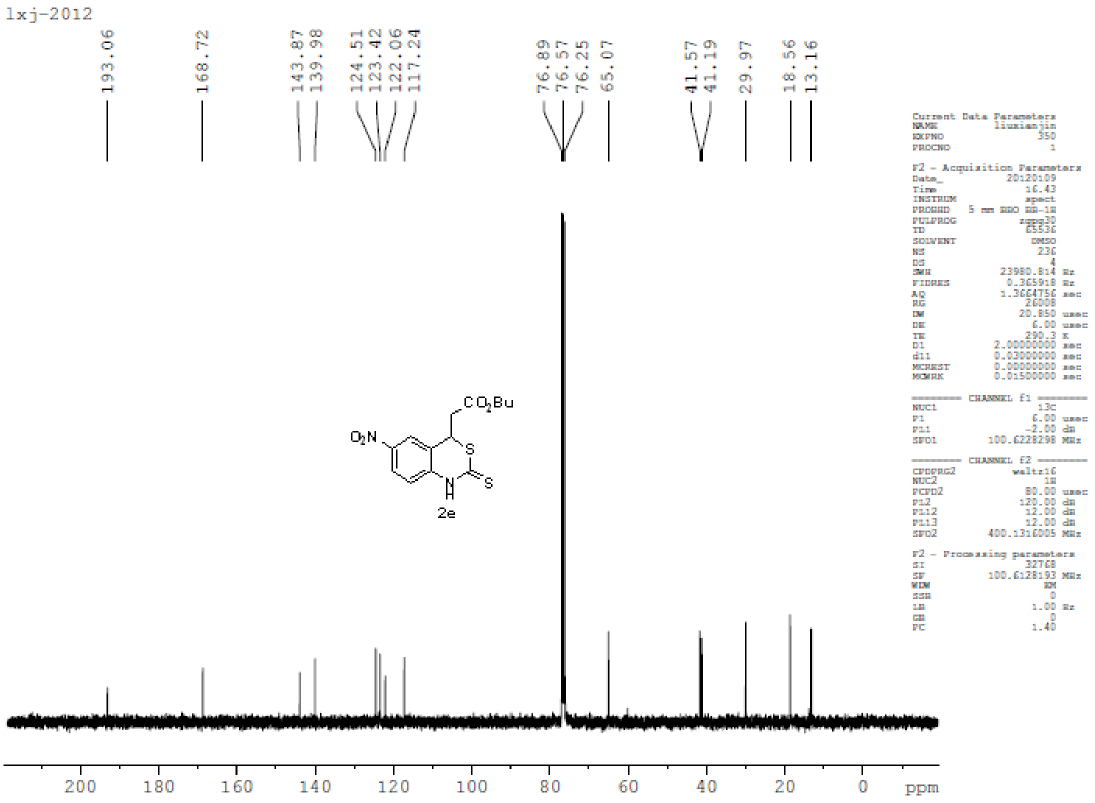

lxj-2012
193.06
168.72
143.87
139.98
124.51
123.42
122.06
117.24
76.89
76.57
76.25
65.07
41.57
41.19
29.97
18.56
13.16
2e
200
180
160
140
120
100
80
60
40
20
0
ppm

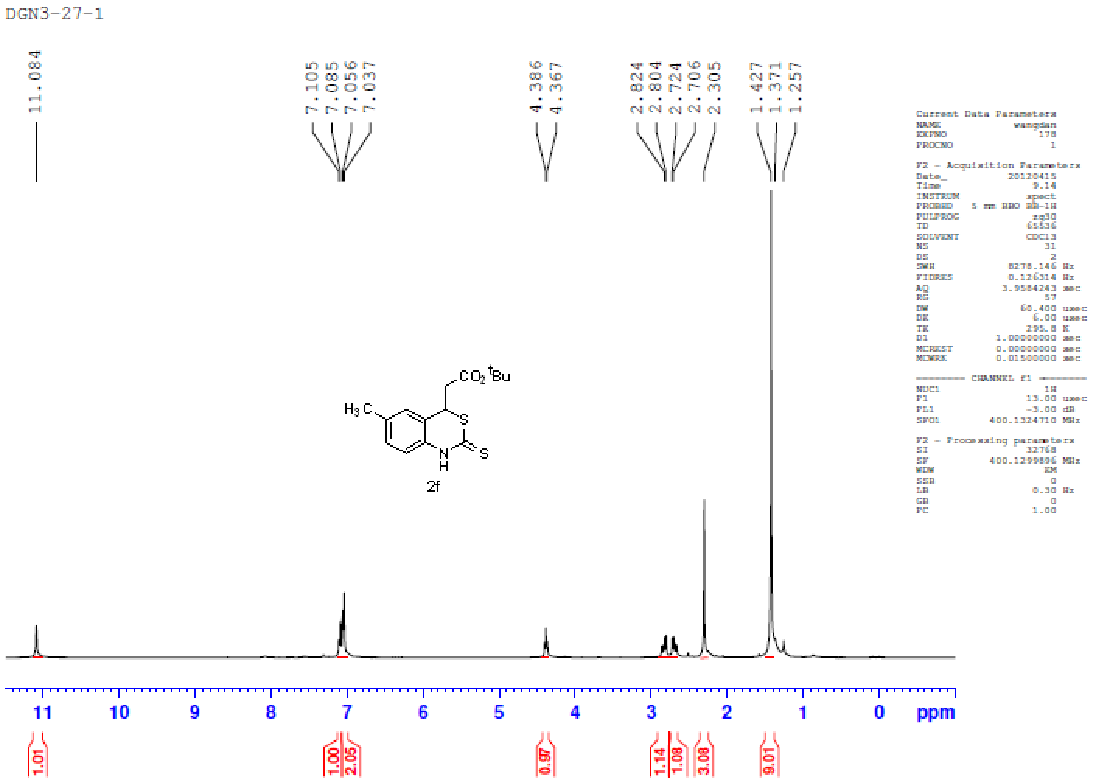
DGN3-27-1
11.084
7.105
7.085
7.056
7.037
4.386
4.367
2.824
2.804
2.724
2.706
2.305
1.427
1.371
1.257
2f
1.01
1.00
2.05
0.97
1.14
1.08
3.08
9.01
ppm

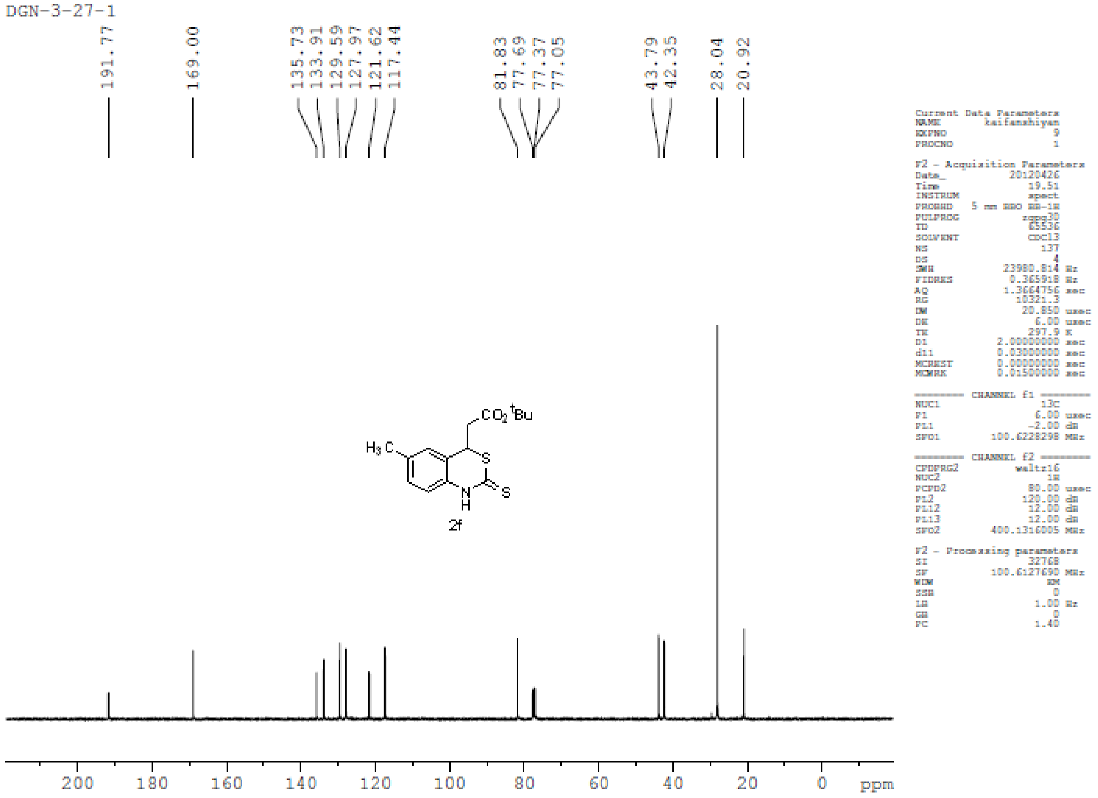
DGN-3-27-1
191.77
169.00
135.73
133.91
129.59
127.97
121.62
117.44
81.83
77.69
77.37
77.05
43.79
42.35
28.04
20.92
2f
ppm

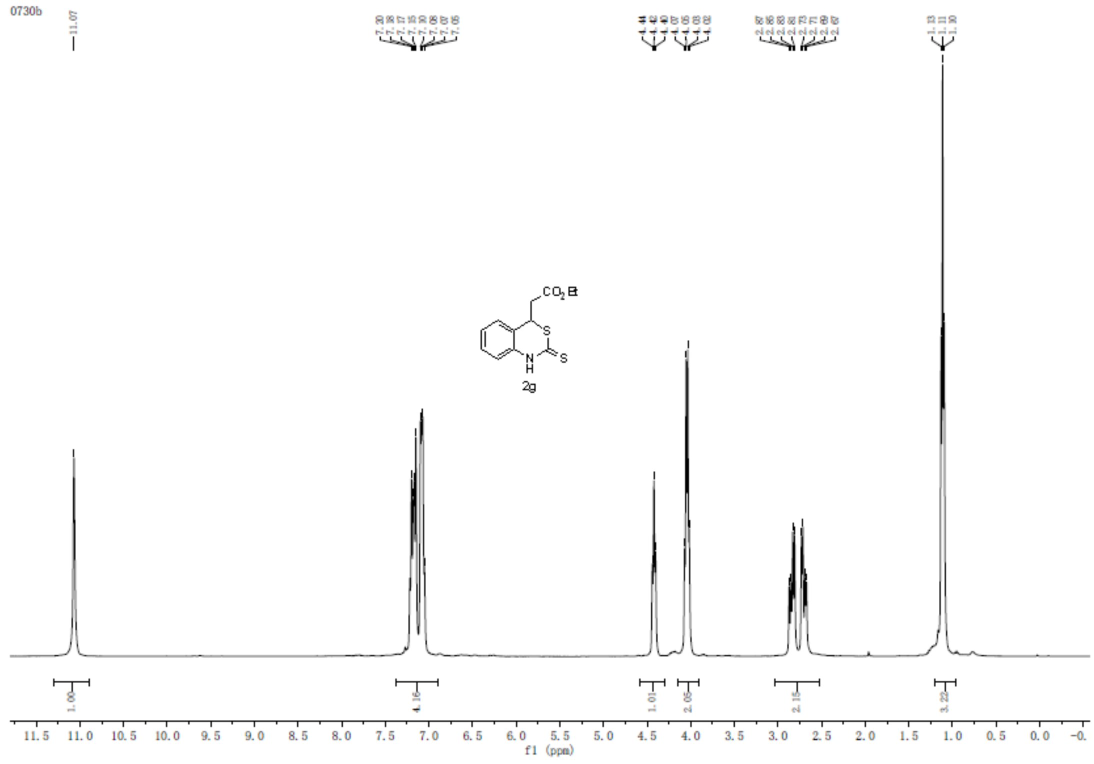
0730b
11.07
CO_2Et
S
N
H
S
2g
1.00
4.16
1.01
2.05
2.15
3.22
f1 (ppm)

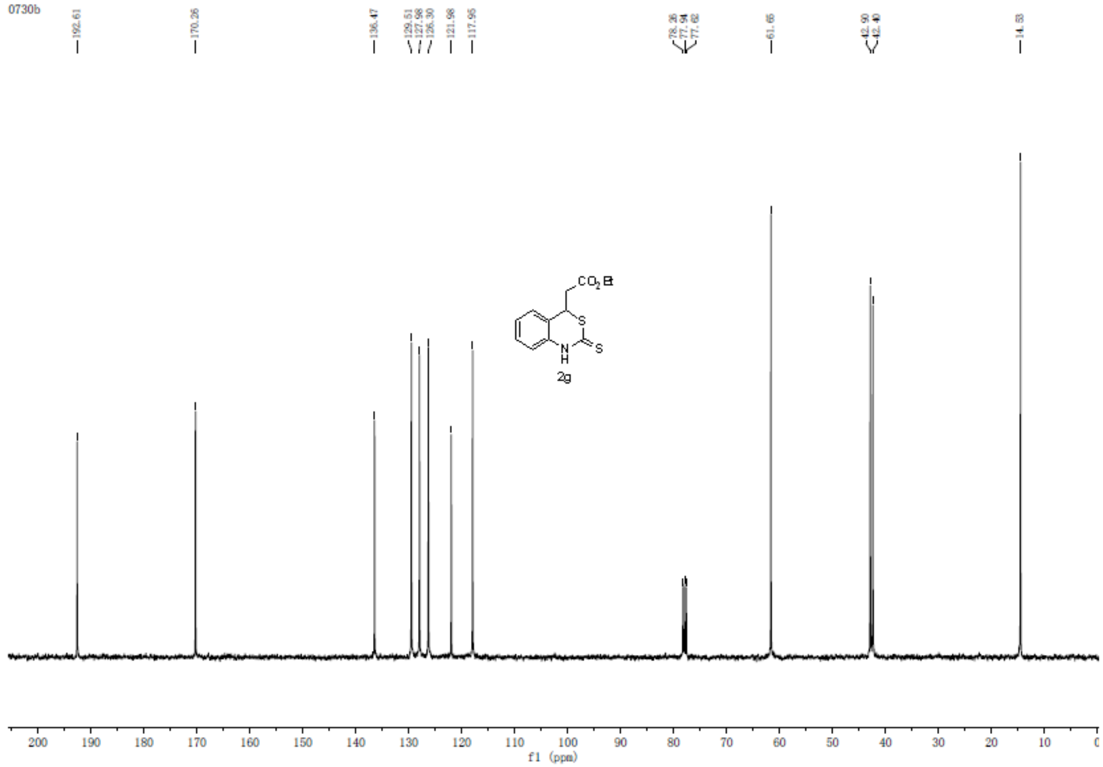
0730b
192.61
170.26
136.47
129.51
127.98
126.30
121.98
117.95
78.26
77.94
77.62
61.65
42.90
42.40
14.53
CO_2Et
S
N
H
S
2g
f1 (ppm)

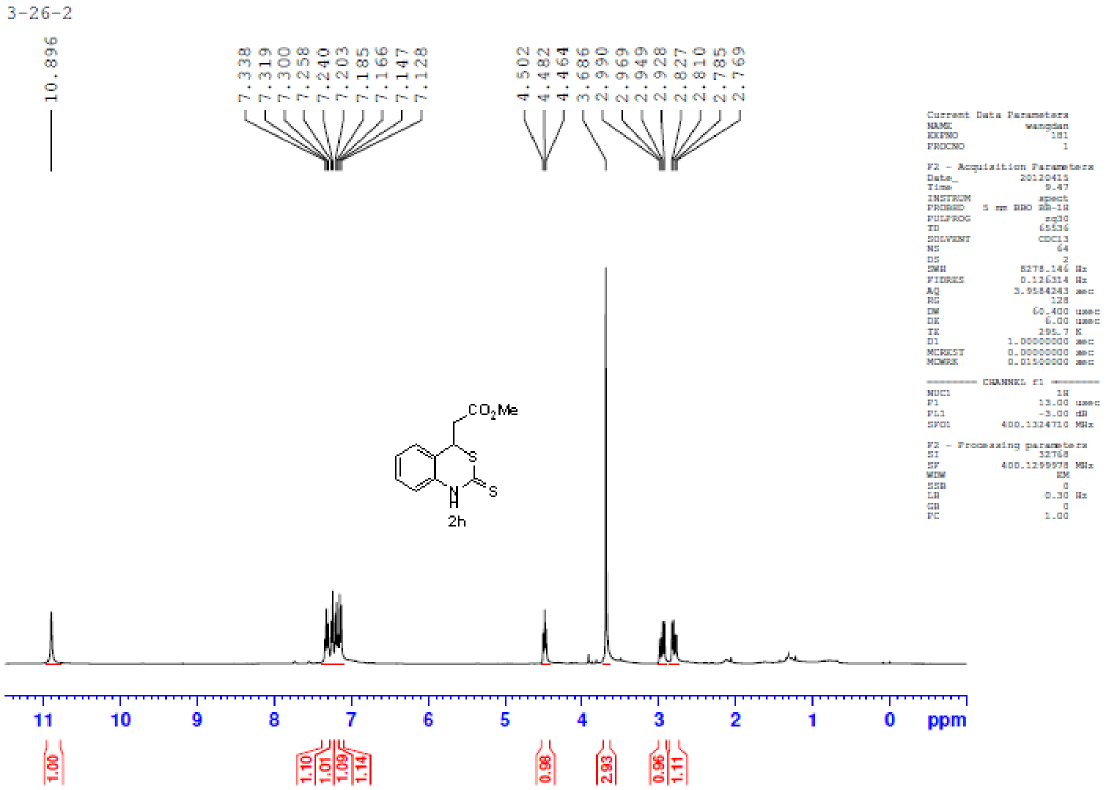
3-26-2
10.896
7.338 7.319 7.300 7.258 7.240 7.203 7.185 7.166 7.147 7.128
4.502 4.482 4.464 3.686 2.990 2.969 2.949 2.928 2.827 2.810 2.785 2.769
CO2Me
S
S
H
N
2h
11 10 9 8 7 6 5 4 3 2 1 0 ppm
1.00 1.10 1.01 1.09 1.14 0.98 2.93 0.96 1.11

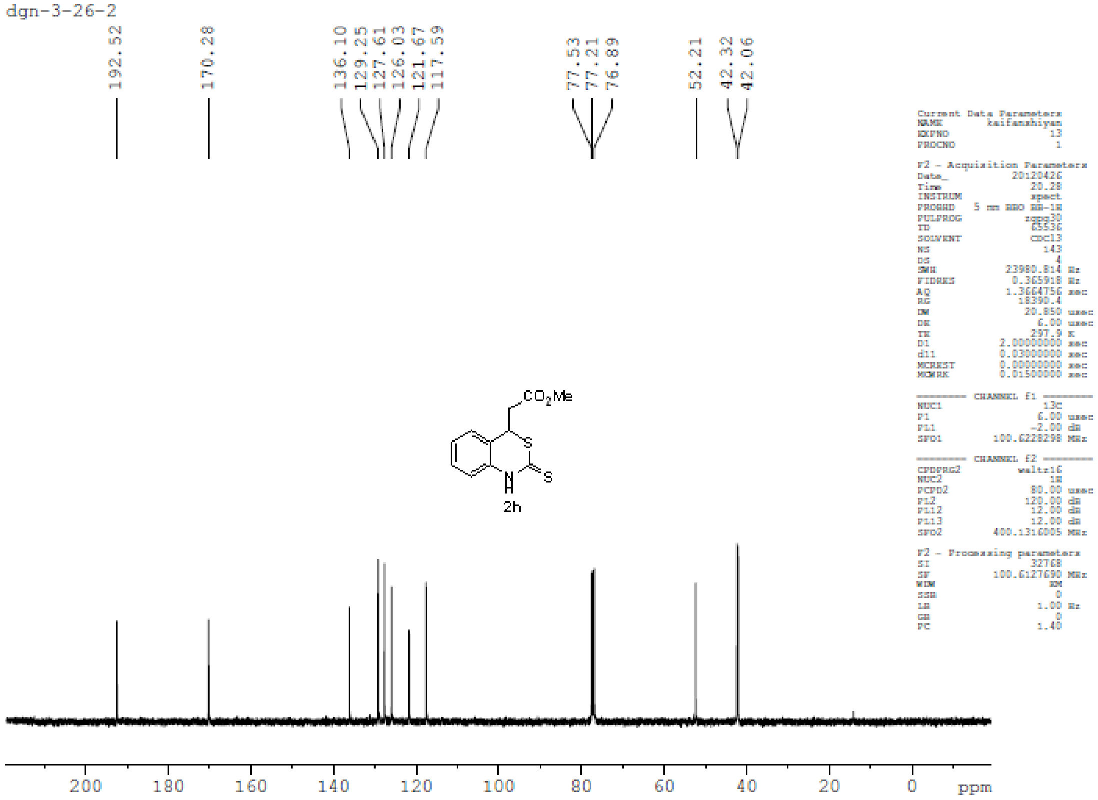
dgn-3-26-2
192.52
170.28
136.10 129.25 127.61 126.03 121.67 117.59
77.53 77.21 76.89
52.21 42.32 42.06
CO2Me
S
S
H
N
2h
200 180 160 140 120 100 80 60 40 20 0 ppm

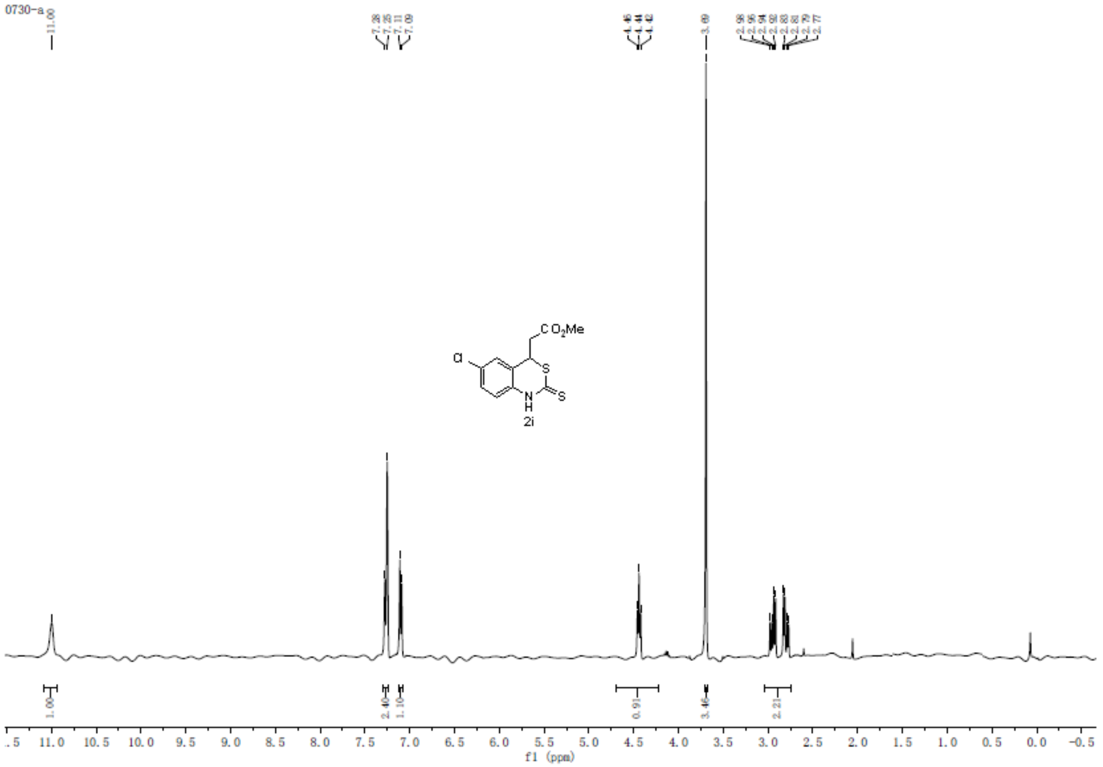
0730-a
11.00
7.28
7.25
7.11
7.09
4.46
4.44
4.42
3.69
1.00
2.40
1.10
0.91
3.46
2.21
f1 (ppm)
CO2Me
Cl
S
N
H
S
2i

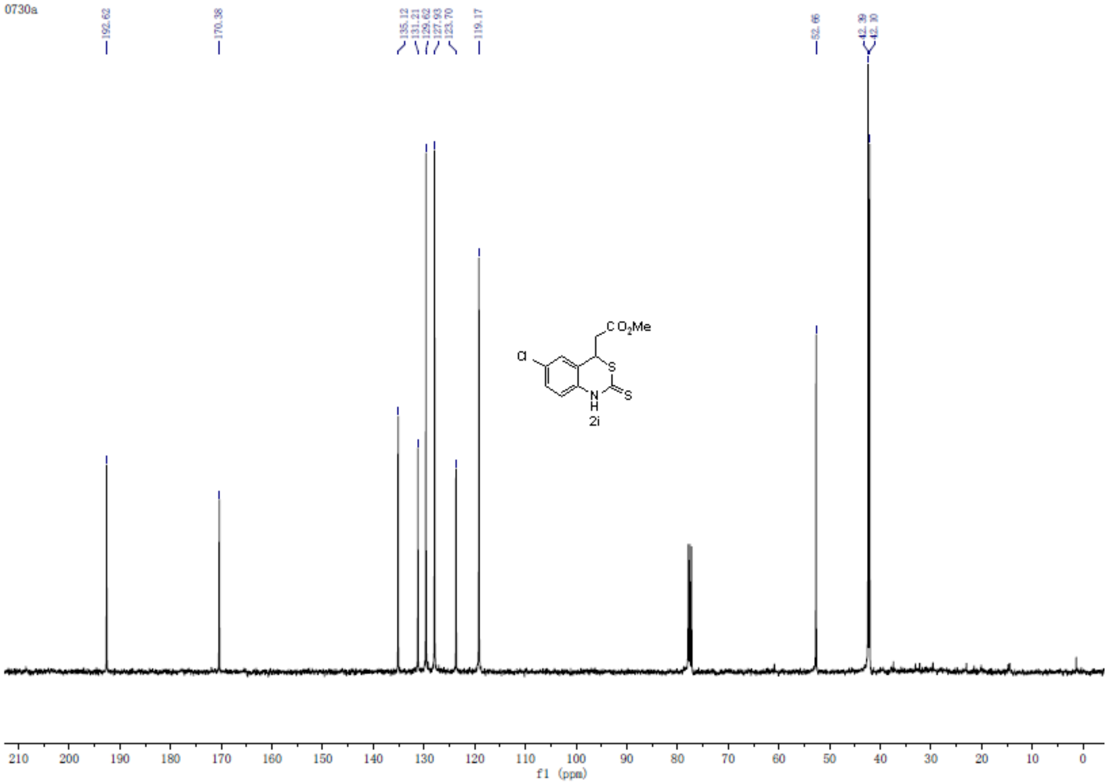
0730a
192.62
170.38
135.12
131.21
129.62
127.93
123.70
119.17
52.66
42.39
42.10
f1 (ppm)
CO2Me
Cl
S
N
H
S
2i

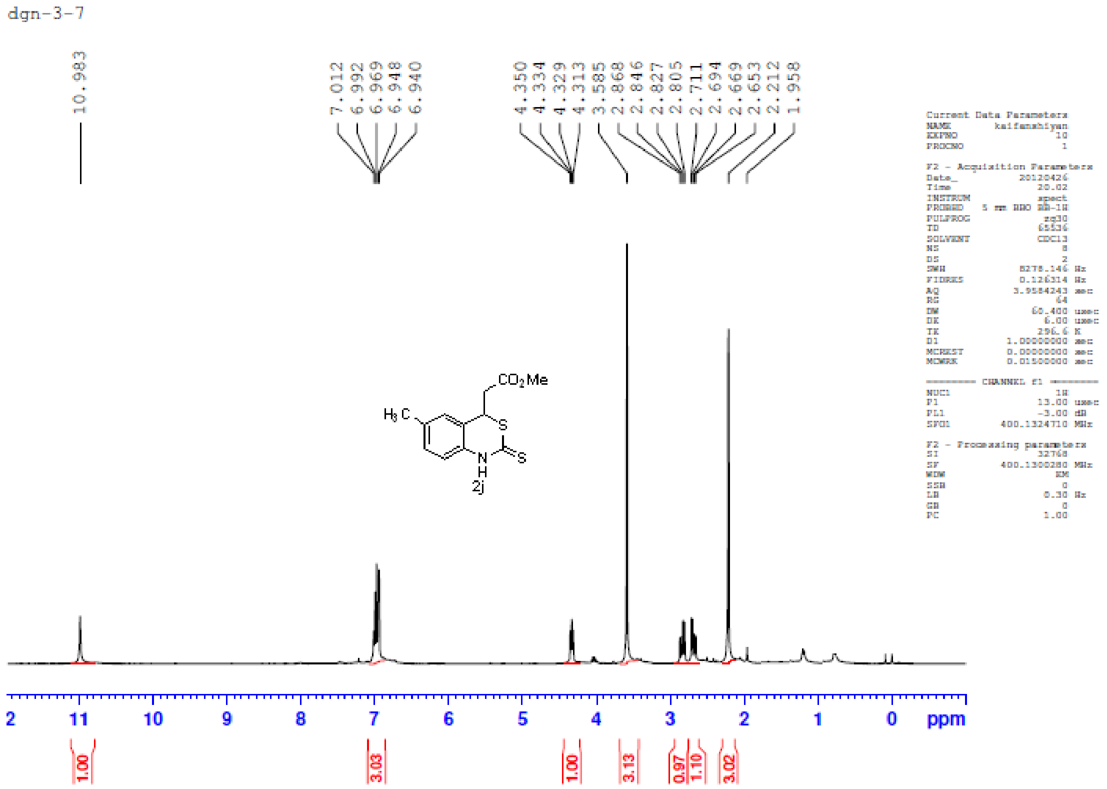
dgn-3-7
10.983
7.012
6.992
6.969
6.948
6.940
4.350
4.334
4.329
4.313
3.585
2.868
2.846
2.827
2.805
2.711
2.694
2.669
2.653
2.212
1.958
CO_2Me
H_3C
S
S
N
H
2j
ppm
1.00
3.03
1.00
3.13
0.97
1.10
3.02

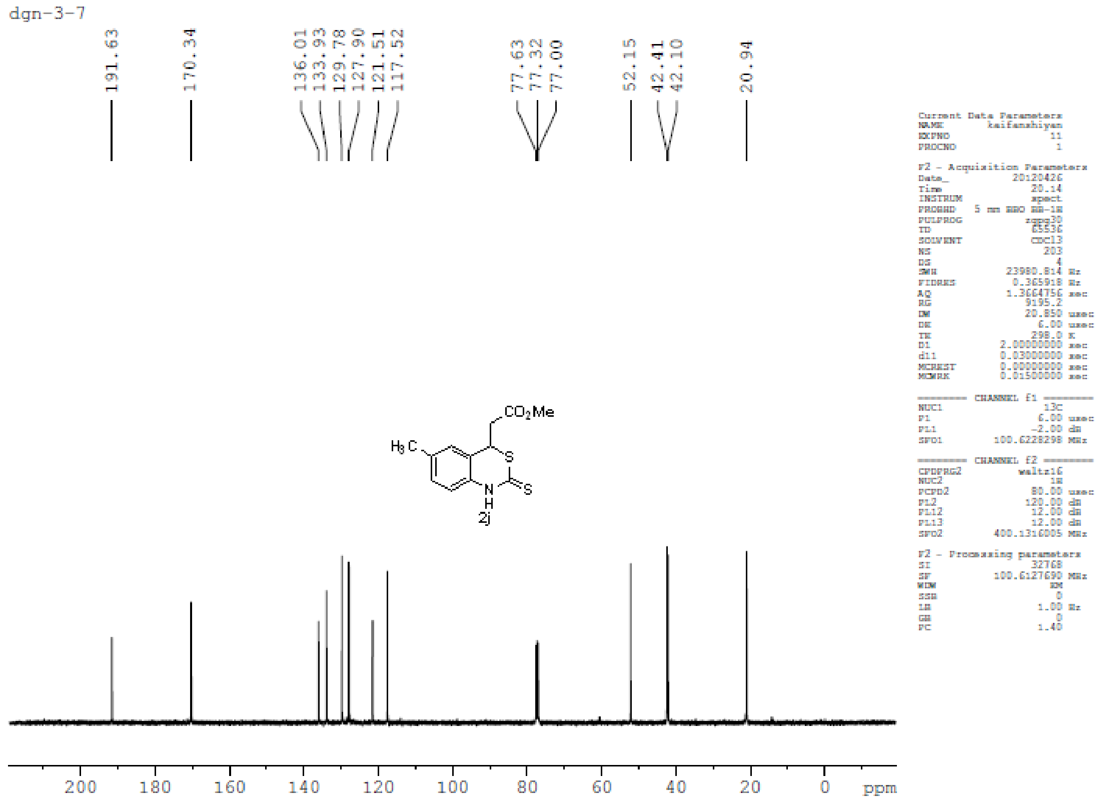
dgn-3-7
191.63
170.34
136.01
133.93
129.78
127.90
121.51
117.52
77.63
77.32
77.00
52.15
42.41
42.10
20.94
CO_2Me
H_3C
S
S
N
H
2j
ppm

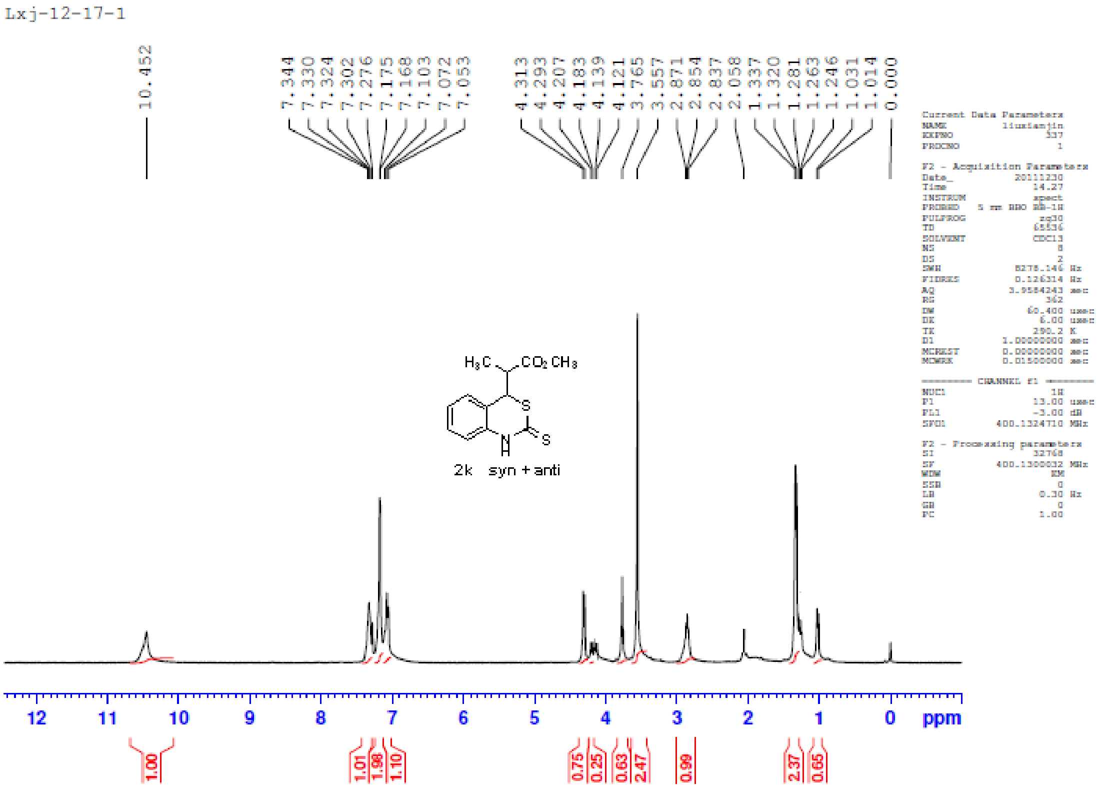
Lxj-12-17-1
10.452
7.344 7.330 7.324 7.302 7.276 7.175 7.168 7.103 7.072 7.053
4.313 4.293 4.207 4.183 4.139 4.121 3.765 3.557 2.871 2.854 2.837 2.058 1.337 1.320 1.281 1.263 1.246 1.031 1.014 0.000
2k syn + anti
ppm
1.00 1.01 1.98 1.10 0.75 0.25 0.63 2.47 0.99 2.37 0.65

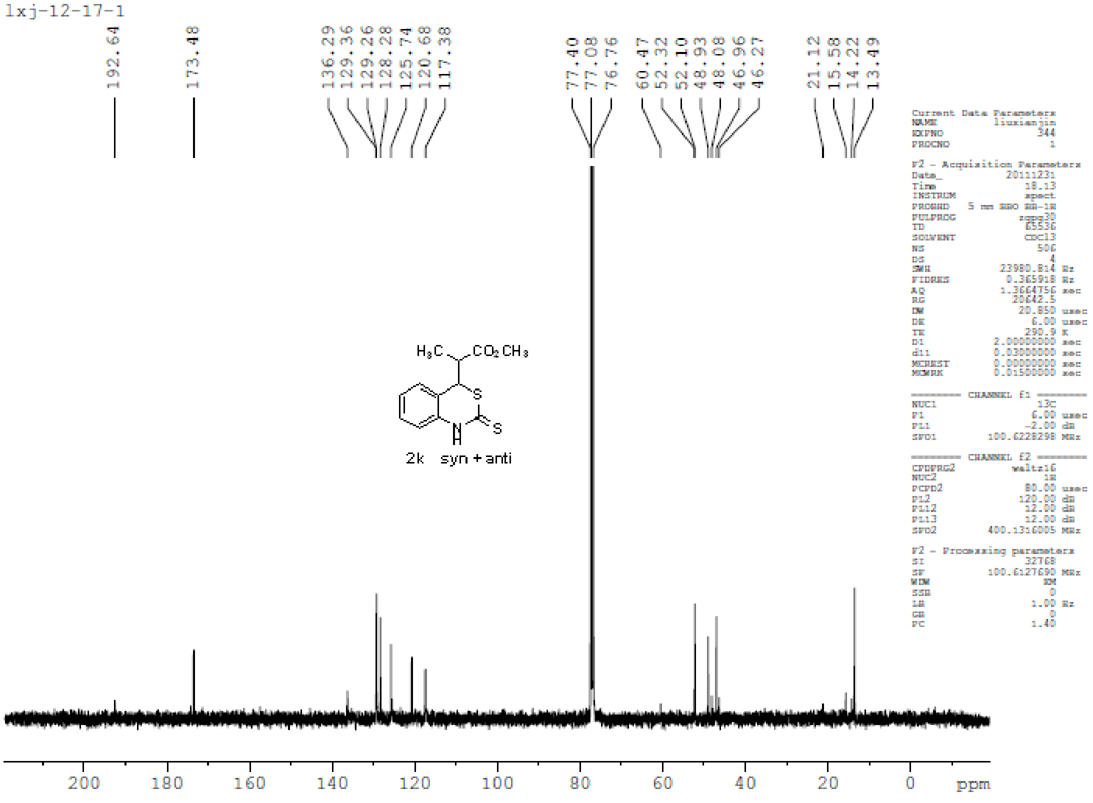
lxj-12-17-1
192.64 173.48 136.29 129.36 129.26 128.28 125.74 120.68 117.38 77.40 77.08 76.76 60.47 52.32 52.10 48.93 48.08 46.96 46.27 21.12 15.58 14.22 13.49
2k syn + anti
ppm

Lxj-12-17-2

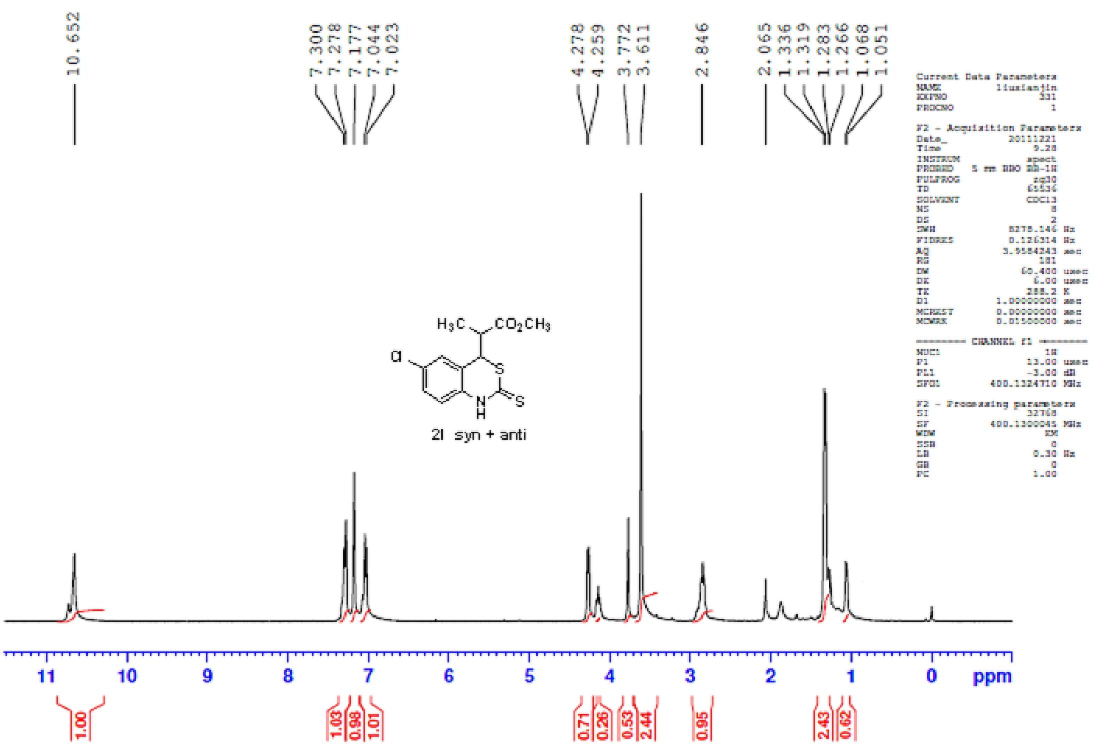

lxj-12-17-2

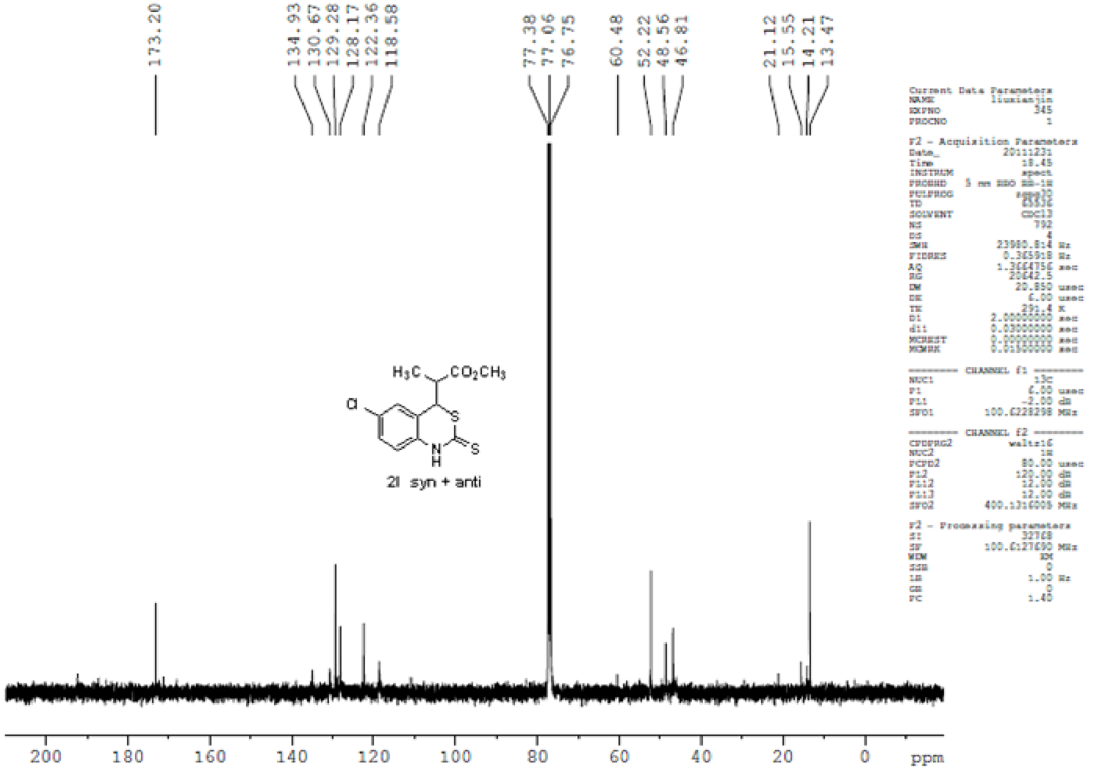

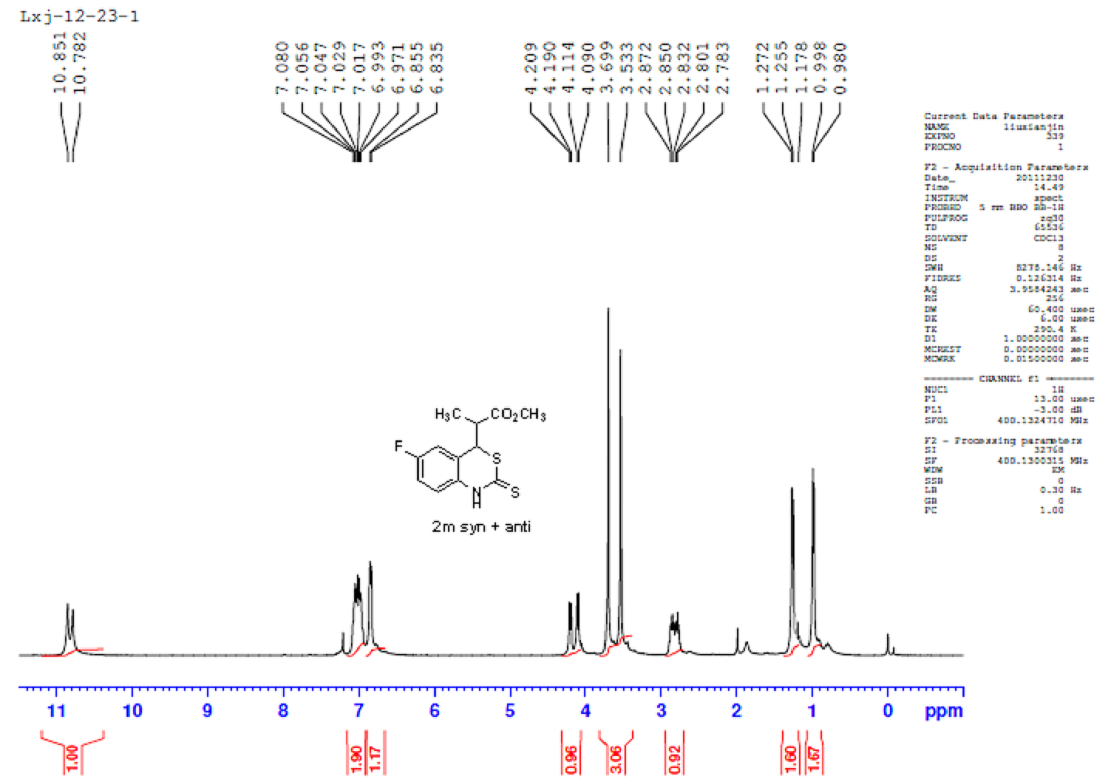
Lxj-12-23-1
10.851
10.782
7.080
7.056
7.047
7.029
7.017
6.993
6.971
6.855
6.835
4.209
4.190
4.114
4.090
3.699
3.533
2.872
2.850
2.832
2.801
2.783
1.272
1.255
1.178
0.998
0.980
2m syn + anti
1.00
1.90
1.17
0.96
3.06
0.92
1.60
1.67
ppm

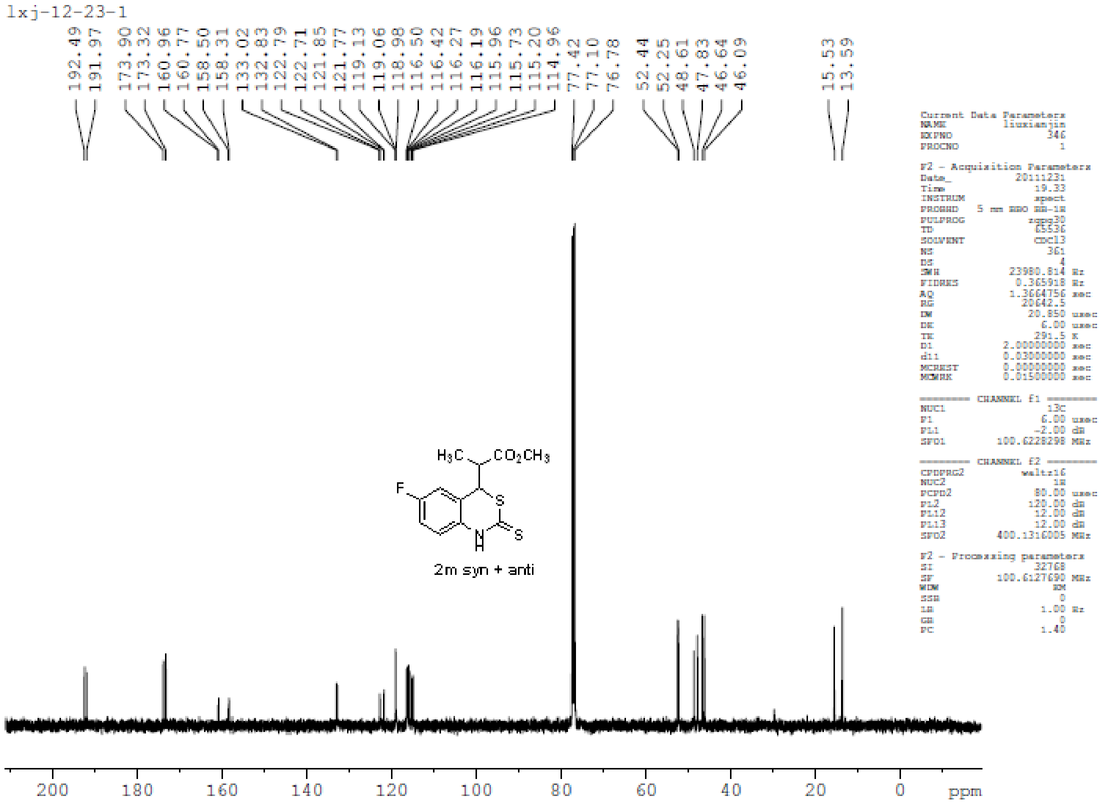
lxj-12-23-1
192.49
191.97
173.90
173.32
160.96
160.77
158.50
158.31
133.02
132.83
122.79
122.71
121.85
121.77
119.13
119.06
118.98
116.50
116.42
116.27
116.19
115.96
115.73
115.20
114.96
77.42
77.10
76.78
52.44
52.25
48.61
47.83
46.64
46.09
15.53
13.59
2m syn + anti
ppm

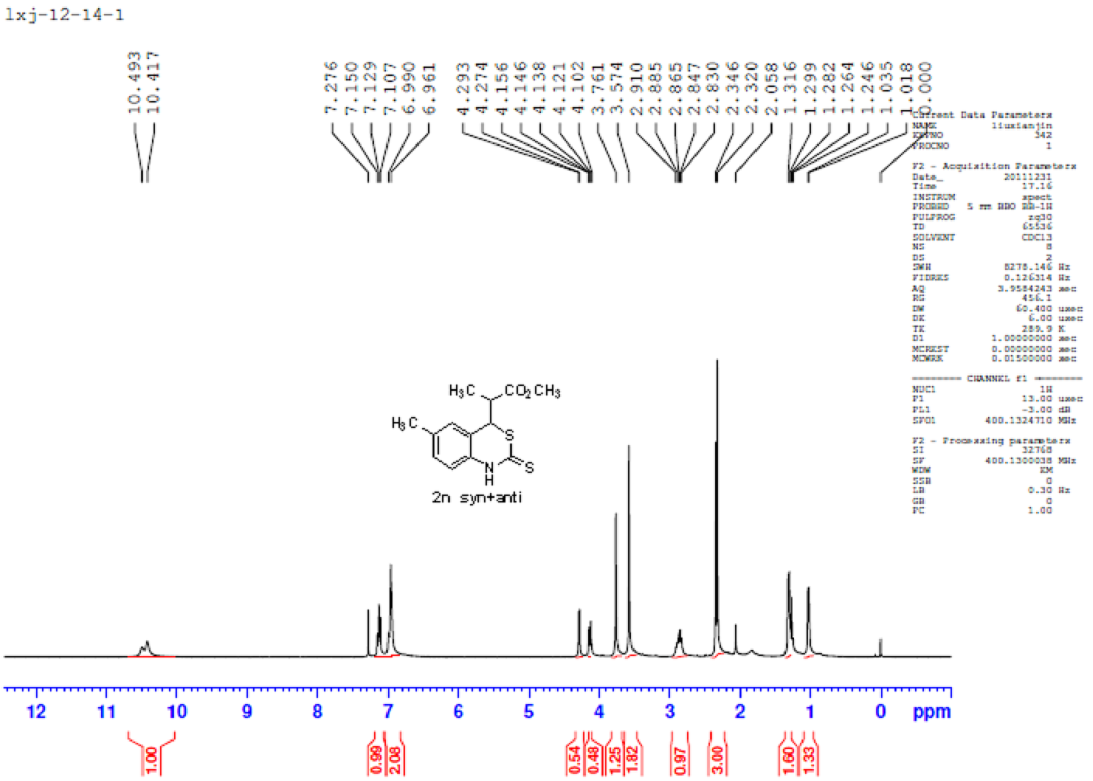
lxj-12-14-1
10.493
10.417
7.276
7.150
7.129
7.107
6.990
6.961
4.293
4.274
4.156
4.146
4.138
4.121
4.102
3.761
3.574
2.910
2.885
2.865
2.847
2.830
2.346
2.320
2.058
1.316
1.299
1.282
1.264
1.246
1.035
1.018
0.000
2n syn+anti
ppm
1.00
0.99
2.08
0.54
0.48
1.25
1.82
0.97
3.00
1.60
1.33

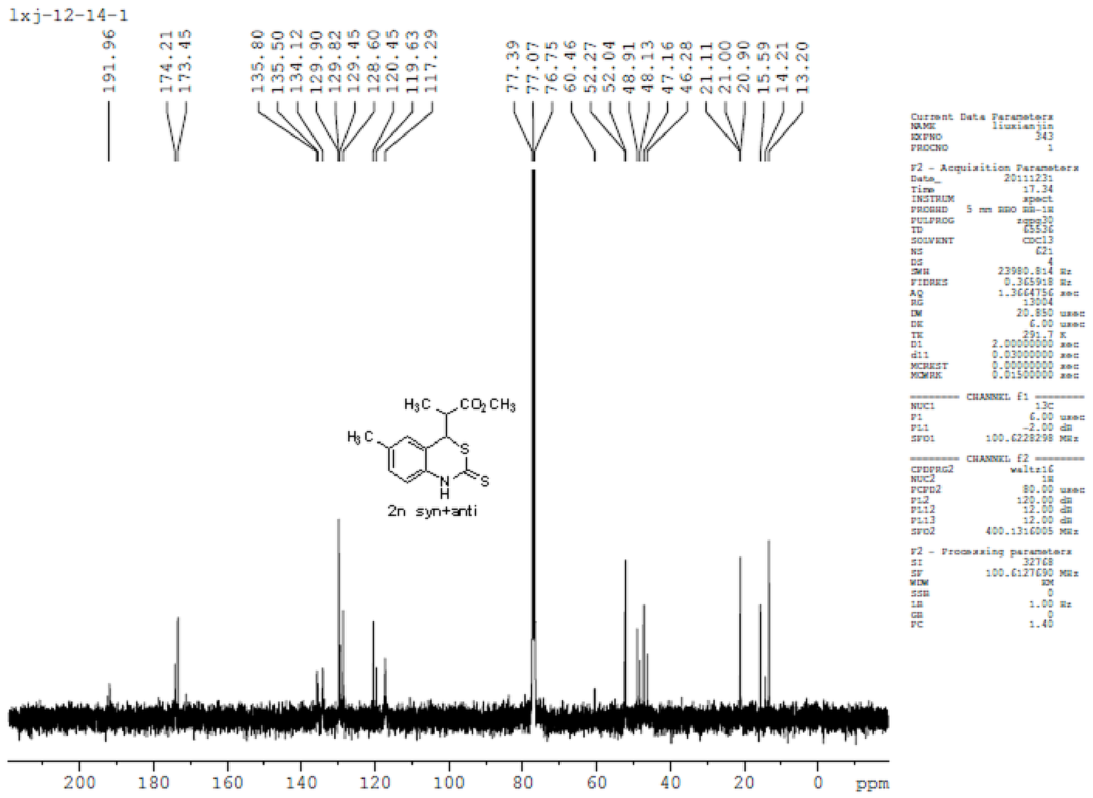
lxj-12-14-1
191.96
174.21
173.45
135.80
135.50
134.12
129.90
129.82
129.45
128.60
120.45
119.63
117.29
77.39
77.07
76.75
60.46
52.27
52.04
48.91
48.13
47.16
46.28
21.11
21.00
20.90
15.59
14.21
13.20
2n syn+anti
ppm

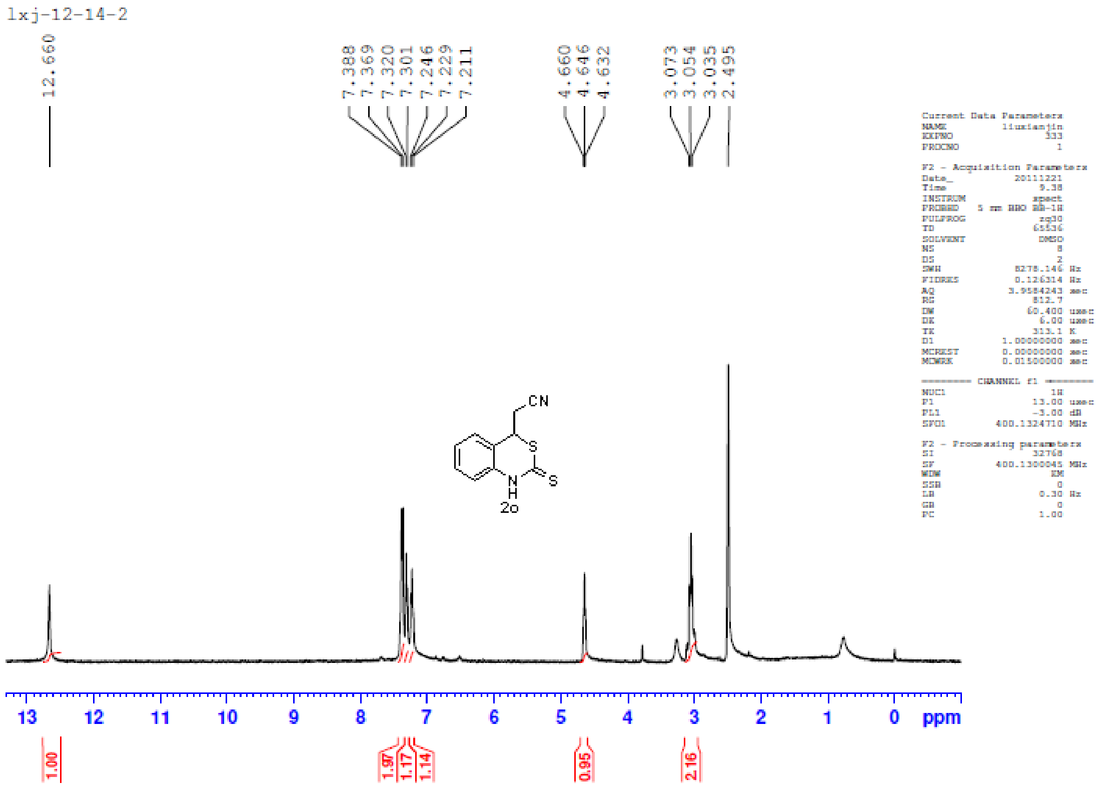
lxj-12-14-2
12.660
7.388 7.369 7.320 7.301 7.246 7.229 7.211
4.660 4.646 4.632
3.073 3.054 3.035 2.495
CN
S
S
N
H
2o
13 12 11 10 9 8 7 6 5 4 3 2 1 0 ppm
1.00
1.97 1.17 1.14
0.95
2.16

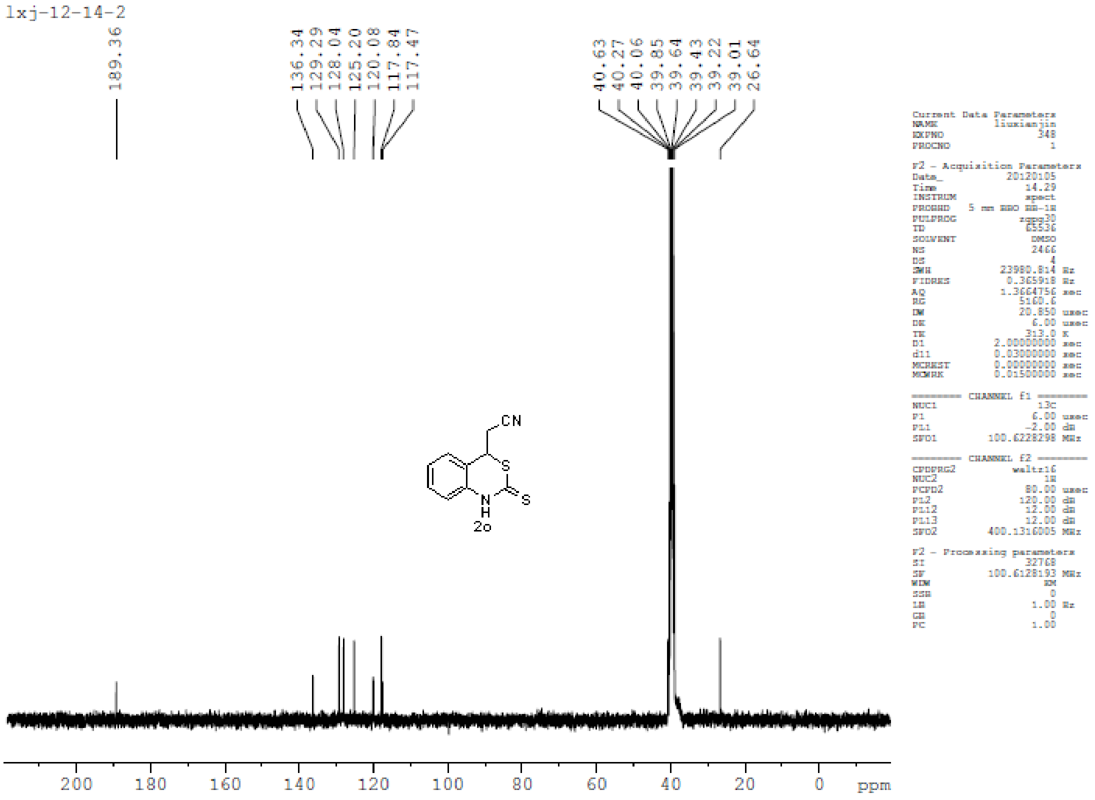
lxj-12-14-2
189.36
136.34 129.29 128.04 125.20 120.08 117.84 117.47
40.63 40.27 40.06 39.85 39.64 39.43 39.22 39.01 26.64
CN
S
S
N
H
2o
200 180 160 140 120 100 80 60 40 20 0 ppm

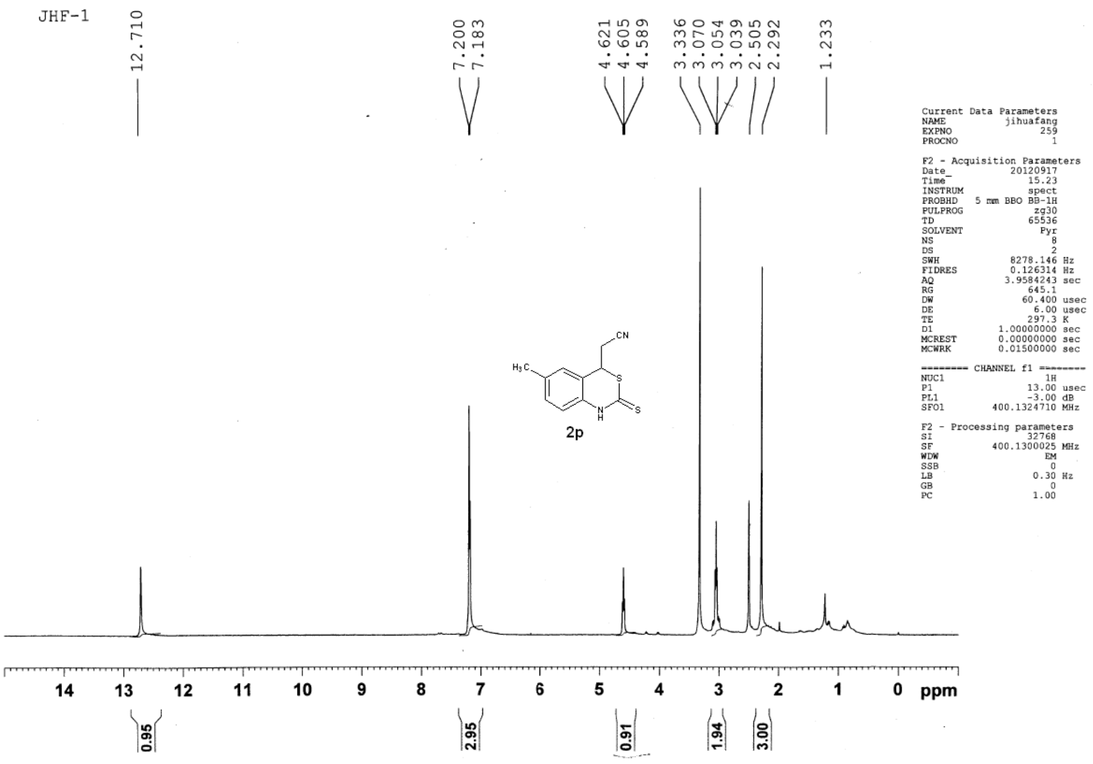
JHF-1
12.710
7.200
7.183
4.621
4.605
4.589
3.336
3.070
3.054
3.039
2.505
2.292
1.233
Current Data Parameters
NAME jihuafang
EXPNO 259
PROCNO 1
F2 - Acquisition Parameters
Date_ 20120917
Time 15.23
INSTRUM spect
PROBHD 5 mm BBO BB-1H
PULPROG zg30
TD 65536
SOLVENT Pyr
NS 8
DS 2
SWH 8278.146 Hz
FIDRES 0.126314 Hz
AQ 3.9584243 sec
RG 645.1
DW 60.400 usec
DE 6.00 usec
TE 297.3 K
D1 1.00000000 sec
MCREST 0.00000000 sec
MCWRK 0.01500000 sec
======== CHANNEL f1 ========
NUC1 1H
P1 13.00 usec
PL1 -3.00 dB
SFO1 400.1324710 MHz
F2 - Processing parameters
SI 32768
SF 400.1300025 MHz
WDW EM
SSB 0
LB 0.30 Hz
GB 0
PC 1.00
CN
H3C
S
N
H
S
2p
14 13 12 11 10 9 8 7 6 5 4 3 2 1 0 ppm
0.95
2.95
0.91
1.94
3.00

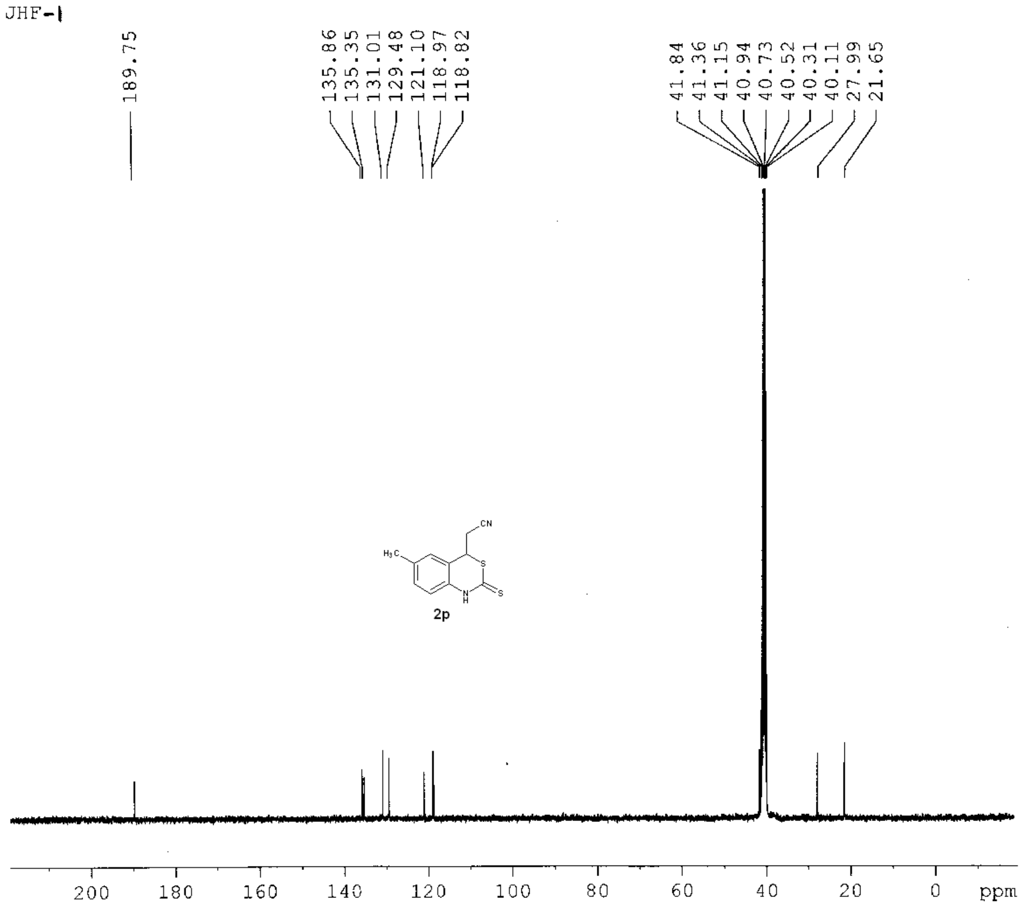
JHF-1
189.75
135.86
135.35
131.01
129.48
121.10
118.97
118.82
41.84
41.36
41.15
40.94
40.73
40.52
40.31
40.11
27.99
21.65
CN
H3C
S
N
H
S
2p
200 180 160 140 120 100 80 60 40 20 0 ppm

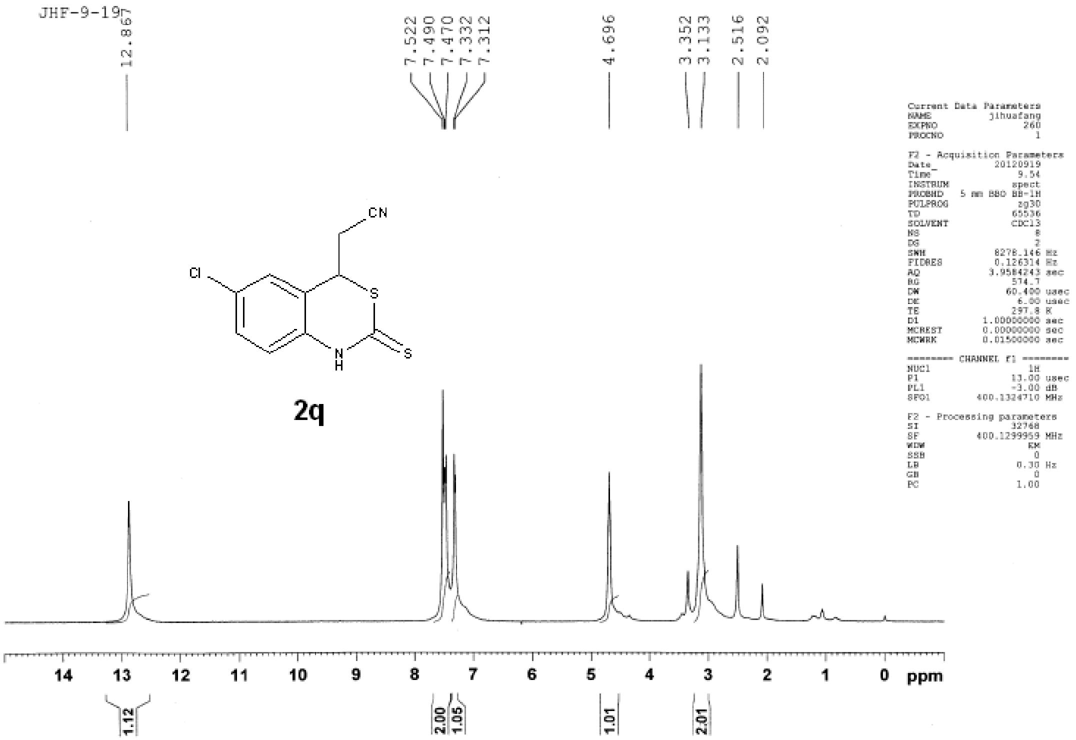
JHF-9-19
12.867
7.522
7.490
7.470
7.332
7.312
4.696
3.352
3.133
2.516
2.092
CN
Cl
S
N
H
S
2q
1.12
2.00
1.05
1.01
2.01
ppm
Current Data Parameters
NAME jihuafang
EXPNO 260
PROCNO 1
F2 - Acquisition Parameters
Date_ 20120919
Time 9.54
INSTRUM spect
PROBHD 5 mm BBO BB-1H
PULPROG zg30
TD 65536
SOLVENT CDCl3
NS 8
DS 2
SWH 8278.146 Hz
FIDRES 0.126314 Hz
AQ 3.9584243 sec
RG 574.7
DW 60.400 usec
DE 6.00 usec
TE 297.8 K
D1 1.00000000 sec
MCREST 0.00000000 sec
MCWRK 0.01500000 sec
======== CHANNEL f1 ========
NUC1 1H
P1 13.00 usec
PL1 -3.00 dB
SFO1 400.1324710 MHz
F2 - Processing parameters
SI 32768
SF 400.1299959 MHz
WDW EM
SSB 0
LB 0.30 Hz
GB 0
PC 1.00

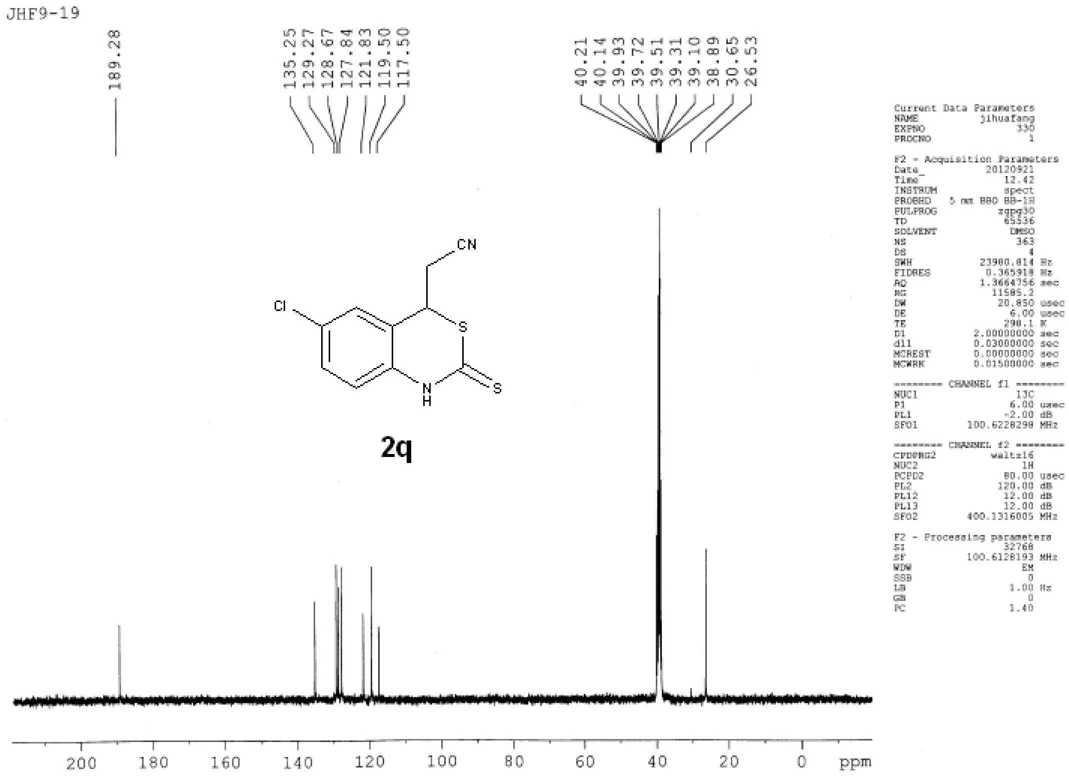
JHF9-19
189.28
135.25
129.27
128.67
127.84
121.83
119.50
117.50
40.21
40.14
39.93
39.72
39.51
39.31
39.10
38.89
30.65
26.53
CN
Cl
S
N
H
S
2q
ppm
Current Data Parameters
NAME jihuafang
EXPNO 330
PROCNO 1
F2 - Acquisition Parameters
Date_ 20120921
Time 12.42
INSTRUM spect
PROBHD 5 mm BBO BB-1H
PULPROG zgpg30
TD 65536
SOLVENT DMSO
NS 363
DS 4
SWH 23980.814 Hz
FIDRES 0.365918 Hz
AQ 1.3664756 sec
RG 11585.2
DW 20.850 usec
DE 6.00 usec
TE 298.1 K
D1 2.00000000 sec
d11 0.03000000 sec
MCREST 0.00000000 sec
MCWRK 0.01500000 sec
======== CHANNEL f1 ========
NUC1 13C
P1 6.00 usec
PL1 -2.00 dB
SFO1 100.6228298 MHz
======== CHANNEL f2 ========
CPDPRG2 waltz16
NUC2 1H
PCPD2 80.00 usec
PL2 120.00 dB
PL12 12.00 dB
PL13 12.00 dB
SFO2 400.1316005 MHz
F2 - Processing parameters
SI 32768
SF 100.6128193 MHz
WDW EM
SSB 0
LB 1.00 Hz
GB 0
PC 1.40

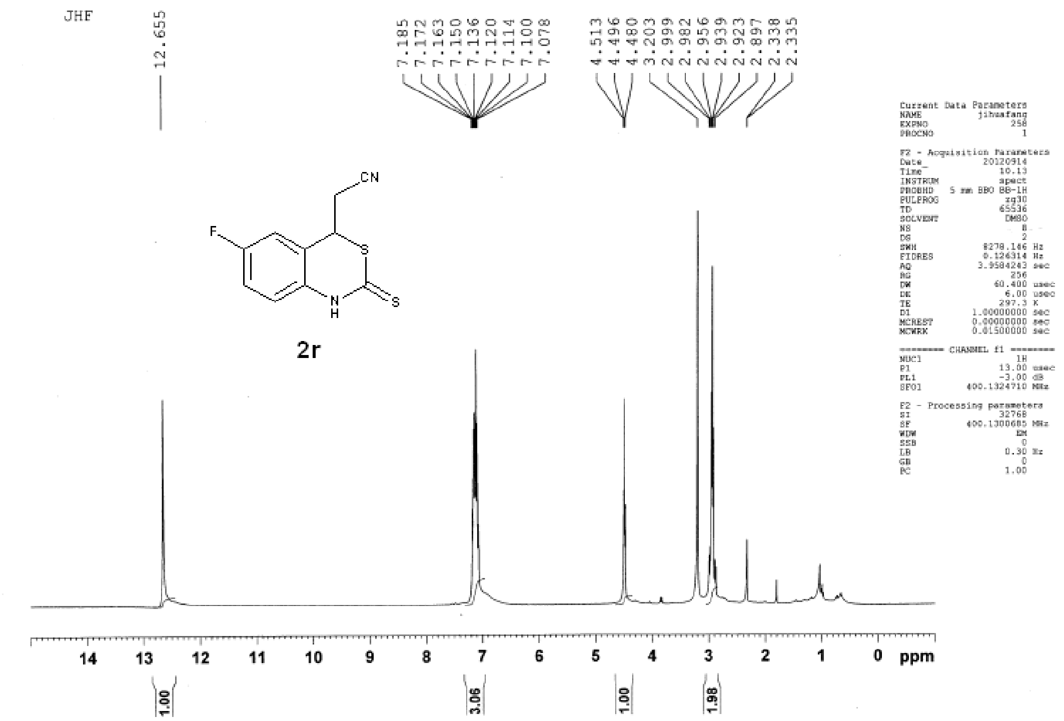
JHF
12.655
7.185 7.172 7.163 7.150 7.136 7.120 7.114 7.100 7.078
4.513 4.496 4.480 3.203 2.999 2.982 2.956 2.939 2.923 2.897 2.338 2.335
2r
1.00
3.06
1.00
1.98
ppm

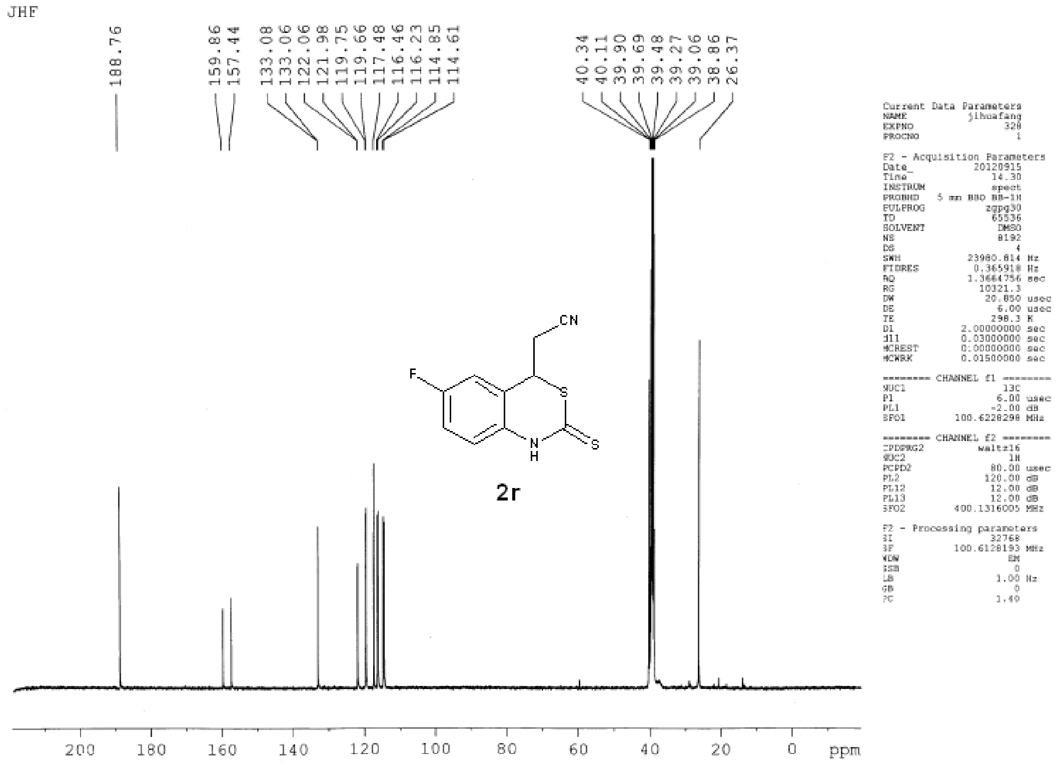
JHF
188.76
159.86 157.44
133.08 133.06 122.06 121.98 119.75 119.66 117.48 116.46 116.23 114.85 114.61
40.34 40.11 39.90 39.69 39.48 39.27 39.06 38.86 26.37
2r
ppm

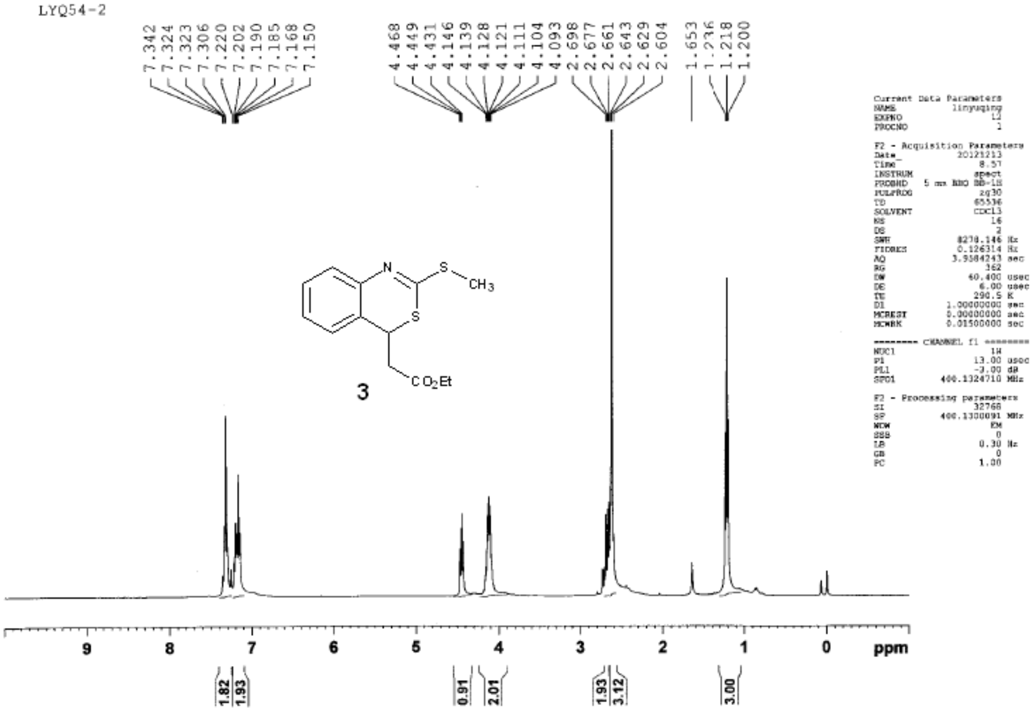
LYQ54-2
7.342
7.324
7.323
7.306
7.220
7.202
7.190
7.185
7.168
7.150
4.468
4.449
4.431
4.146
4.139
4.128
4.121
4.111
4.104
4.093
2.698
2.677
2.661
2.643
2.629
2.604
1.653
1.236
1.218
1.200
3
CO2Et
ppm
1.82
1.93
0.91
2.01
1.93
3.12
3.00

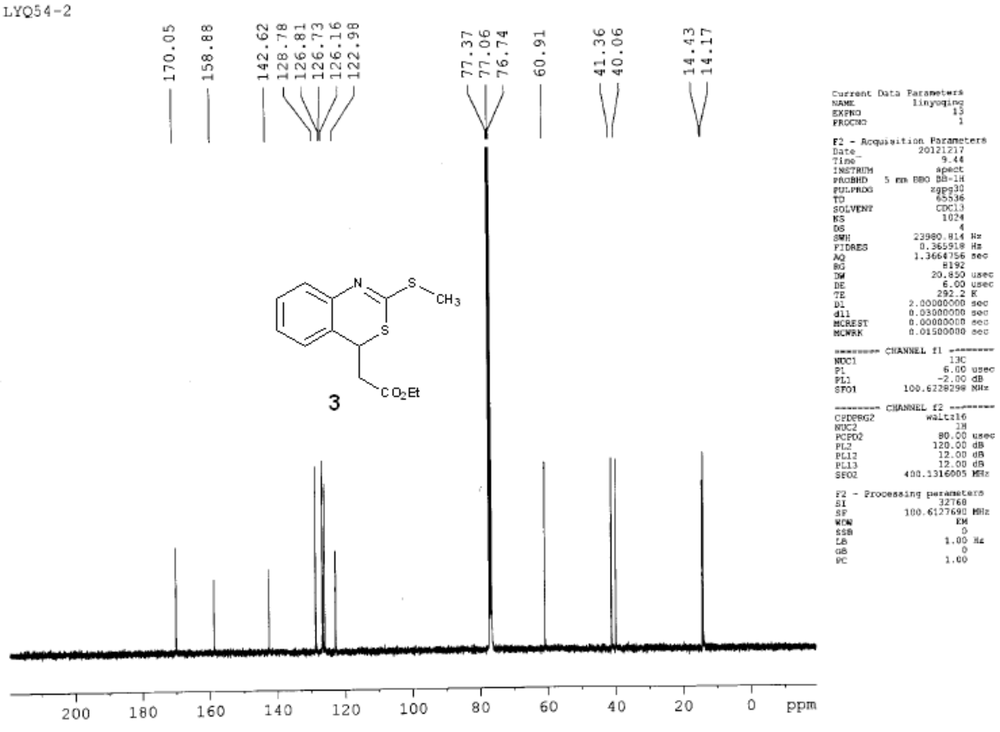
LYQ54-2
170.05
158.88
142.62
128.78
126.81
126.73
126.16
122.98
77.37
77.06
76.74
60.91
41.36
40.06
14.43
14.17
3
CO2Et
ppm
